# Genome-wide identification and expression profile analysis of TALE superfamily genes under hormone and abiotic stress in maize (*Zea may* L.)

**DOI:** 10.3389/fpls.2025.1489177

**Published:** 2025-02-21

**Authors:** Peiyan Guan, Dongbo Zhao, Longxue Wei, Peipei Cui, Shicai Zhang

**Affiliations:** ^1^ College of Life Science, Dezhou University, Dezhou, Shandong, China; ^2^ Food Crops Research Institute, Dezhou Academy of Agricultural Science, Dezhou, Shandong, China; ^3^ Department of Food Testing, Binzhou Testing Center, Binzhou, Shandong, China

**Keywords:** *ZmTALE* transcription factors, maize, genome-wide analysis, hormone response, abiotic stress response, subcellular localization analysis

## Abstract

**Introduction:**

The three-amino-acid-loop-extension (TALE) of the homeobox superfamily genes plays important roles in plant growth, development, and responses to environmental stress. Although TALE members have been identified in various species, they have not been systematically characterized in maize and their expression profiles under ABA hormone and abiotic stress are unknown.

**Methods:**

Bioinformatics methods were employed to identify the TALE family genes in the maize genome. The expression levels of *ZmTALEs* under ABA, salt, drought, and high temperature conditions was detected by qRT-PCR. The subcellular localization of ZmKNOX05 and ZmBELL11 proteins was observed in maize protoplasts.

**Results:**

In this study, we identified 52 TALE members in maize, which can be divided into two subfamilies, KNOX and BELL. ZmKNOXs and ZmBELLs can be further divided into two subclasses based on the domains they contain. The protein characterizations and gene structures in the same subclass were similar, whereas they were distinct across different subclasses. There were 18 collinear gene pairs in maize genome. Inter-species evolutionary analyses showed that TALE family genes of maize were more homologous to monocotyledons than to dicotyledons. The promoter regions of *ZmTALE* contained abundant stress-responsive, hormone-responsive, light-responsive, and plant growth and development cis-elements. Specific spatiotemporal expression patterns analysis showed that *ZmBELLs* were highly expressed in root and mature leaf, whereas the *ZmKNOX1* subfamily genes were more expressed in the primordium, internode, vegetative meristem, and root during developmental stages. It was found that most *ZmTALEs* could respond to ABA, drought, high temperature, and salt stress, indicating their roles in hormone and abiotic stress responsive. *ZmKNOX05* and *ZmBELL11* were cloned from B73 maize. Unexpectedly, a novel alternative transcript with a 99-base deletion for *ZmKNOX05* were found, named ZmKNOX05.2, which exhibited alternative splicing event at the noncanonical site. Subcellular localization analysis revealed that ZmKNOX05.1-eGFP and ZmKNOX05.2-eGFP were localized in both the nucleus and cytoplasm, while ZmBELL11-eGFP was localized in perinuclear cytoplasm (perinuclear region of the cytoplasm).

**Discussion:**

We identified TALE superfamily members in maize and conducted a comprehensive and systematic analysis. These results can lay the foundation for analysis of the functions of *ZmTALE* genes under ABA and abiotic stresses.

## Introduction

Homeobox transcription factors contain a highly conserved DNA-binding domain of 60 amino acids, which is encoded by a 180 bp homeobox DNA sequence, called homeobox domain (HD). HD consists of three α-helices, with a ring between the first and the second helix, and a helix-turn-helix structure in the second and third α-helices. The homeobox gene was first discovered in *Drosophila* melanogaster during the study of homeotic mutants, that produce homeotic phenotypes when mutated (Mcginnis et al., 1984a, 1984b; Scott and Weiner, 1984). Subsequently, the first plant homeobox gene *KNOTTED-1* (Kn1) was identified in maize through transposon tagging in 1991 (Vollbrecht et al., 1991). Based on sequence features, the homeobox transcription factor were divided into many different classes. They can be divided into seven classes by Bharathan, including knotted-like homeobox (KNOX/KNAT), BEL1-like homeobox (BELL/BLH), *Zea mays* homeobox (ZM-HOX), homeobox from *A. thaliana 1* (HAT1), homeobox from *A. thaliana 2* (HAT2), *Arabidopsis thaliana* homeobox 8 (ATHB8), and GL2 (Bharathan et al., 1997). And were divided into five classes, including HD-ZIP, GLABRA, KNOTTED, PHD, and BEL by Chan (Chan et al., 1998). Later, Bürglin and Affolter divided them into 11 classes, including HD-ZIP (with four subclasses: I to IV), wuschel-related homeobox (WOX), nodulin homeobox (NDX), plant homeodomain (PHD), plant zinc finger (PLINC), luminidependens (LD), DDT, SAWADEE, PINTOX, KNOX and BEL (Burglin and Affolter, 2016). The three-amino-acid-loop-extension (TALE) family genes, including KNOX and BELL, encode atypical homeodomain proteins, which contain three extra conserved amino acids “PYP” (proline-tyrosine-proline) between the first and second helices of the homeodomain (Burglin, 1997).

The KNOX transcription factors usually contain four characteristic domains: KNOX1, KNOX2, ELK, and HD: with the exception of the KNATM family (Gao et al., 2015). The KNOX1 and KNOX2 domains together are also known as MEINOX (Myeloid ecotropic viral integration site and KNOX) domain (Hamant and Pautot, 2010; Niu and Fu, 2022). According to sequence similarity, evolutionary features and expression patterns, KNOX proteins were divided into three classes in Arabidopsis (Gao et al., 2015). The class I of KNOX consists four genes in Arabidopsis, *SHOOTMERISTMELESS* (STM), *KNAT1*, *KNAT2*, and *KNAT6*. They were mainly expressed in meristem, stem and leaf primordia, and played important roles in the diversity of leaf form (Hake et al., 2004). In Arabidopsis, the *stm* loss-of-function mutants do not form a shoot apical meristem (SAM) (Long et al., 1996). The Arabidopsis KNOX II class contains four members: *KNAT3*, *KNAT4*, *KNAT5*, and *KNAT7*, which were expressed in the inflorescence stems, and played important roles in secondary cell wall (SCW) formation (Qin et al., 2020; Wang et al., 2020). In addition, KNATs play functionally redundant roles in *Arabidopsis thaliana* root and seed coat development (Dean et al., 2004; Truernit and Haseloff, 2007; Bhargava et al., 2013). The third class of KNOX proteins includes KNATM, members of which were found only in dicotyledons. KNATM can interact with other TALE members to regulate their activities, and influence leaf polarity and development (Magnani and Hake, 2008).

BEL1-like homeobox transcription factors have conserved SKY and BELL domain at the N-terminus (also known as MID or POX domain) upstream of the HD. And some members have a “VSLTLGL” sequence at the C-terminus with unknown functions (Niu and Fu, 2022). BELL transcription factors play important roles in many aspects of plant growth, and development (Niu and Fu, 2022). In *Arabidopsis thaliana*, loss-of-function of *BELL-1* causes transformation of ovule integuments into carpels via negative regulation of *AGAMOUS* (Reiser et al., 1995). Overexpression of the light-regulated *ARABIDOPSIS THALIANA HOMEOBOX1* (ATH1) induces delayed flowering through positive regulation of FLC (Proveniers et al., 2007). BLH12 and BLH14 was involved in the maintenance of maize meristem by interacting with knottted1 (KN1) (Tsuda et al., 2017).

TALE family members have been identified in many species, such as Arabidopsis (Hamant and Pautot, 2010), poplar (Zhao et al., 2019), cotton (Ma et al., 2019), *Prunus mume* (Yang et al., 2022), *Triticum aestivum* (Han et al., 2022; Rathour et al., 2022), barley (Liao et al., 2024), and soybean (Wang et al., 2021). They were found not only play important roles in plant growth and development (Hamant and Pautot, 2010; Ma et al., 2019; Rathour et al., 2022), but also are involved in hormone regulatory pathways and responses to various environmental stresses (Zhao et al., 2019; Wang et al., 2021; Han et al., 2022; Rathour et al., 2022; Yang et al., 2022; Liao et al., 2024). In recent years, the roles of TALE family genes in response to environmental stresses (e.g. drought, salinity, heat, ABA hormones, etc.) have sparked the attention of researchers. For example, in wheat, the expression of most of the BELL genes are down-regulated and most of the KNOX genes are up-regulated under heat and drought stress conditions. Overexpression of *TaKNOX11* enhances drought and salt stress resistance in *Arabidopsis thaliana* (Han et al., 2022). In soybean, many *GmTALEs* can response to salt stress and dehydration in leaf, stem and root tissues (Wang et al., 2021). In poplar, out of the 35 TALE genes, 11 genes are responsive to salt stress (Zhao et al., 2019). Most *HvTALEs* can response to multiple exogenous hormones in barley (Liao et al., 2024). These studies indicated that the TALE transcription factors play important roles in plant response to adverse environments.

Maize is an important food-cum-cash crop across the globe. It is susceptible to extreme weather, such as drought, high temperature, and salinity during its growth and developmental process. And adversely environment seriously affect maize production. Given that the TALE family has important roles in response to environment stress and little is known about the function of TALE genes under abiotic stress in maize. In this study, we identified the TALE family members in maize through genome-wide screening and analyzed their chromosomal localization, evolutionary relationships, promoter cis-elements and expression characteristics. Meanwhile, qRT-PCR was used to analyze the expression patterns of 13 genes under drought, salt, high temperature, and ABA hormone conditions. Finally, we observed the subcellular localization of KNOX05 and BELL11 in maize protoplasts using laser confocal microscopy. These results will lay the foundation for regulation and function analyses of the maize TALE gene family by hormone and abiotic stress during plant growth and development, and enrich the gene resources for molecular breeding in maize.

## Materials and methods

### Plant materials and treatments

The seeds of maize inbred line B73, were cultivated in a growth chamber under long day conditions 16h of light, 25°C, and 8h of darkness, 22°C. These conditions were consistently maintained throughout the entire experimental period. For drought and salt stress treatments, the B73 seedlings at the three-leaf stage were watered either with 35% PEG-6000 (w/v), or 200 mM NaCl (Cai et al., 2017). Heat stress was applied by transferring the seedlings to plant growth cabinet at 42°C (Shi et al., 2017). For hormone treatment, seedlings were sprayed with 100 µM ABA (Cai et al., 2017). Leaf samples were harvested at the designated times and snap-frozen for all applicable treatments, then stored at -80°C for RNA extraction.

### Identification and characterization of TALE gene family members in maize

The protein sequences of maize TALE family were retrieved from Plant TFDB (http://planttfdb.gao-lab.org/) (Jin et al., 2017). These proteins were viewed as candidates of TALE family members. Then the sequences were submitted to Pfam (http://pfam.xfam.org/) (Mistry et al., 2021) and NCBI Batch CD-Search (https://www.ncbi.nlm.nih.gov/Structure/bwrpsb/bwrpsb.cgi) for verification (Wang et al., 2023), to ensure that all proteins contained the conserved structural domains of TALE family. Proteins containing HD and POX domains were considered as BELL family, and proteins with HD and KNOX conserved domains were considered as KNOX family. Ultimately, we acquired 52 TALE genes in maize. The ProtParam tool (https://web.expasy.org/protparam/) was utilized to obtain basic information related to protein characterization, including molecular weight, theoretical pI, instability index, grand average of hydropathicity (GRAVY), aliphatic index and other parameters (Wilkins et al., 1999). Their molecular weight, protein length, and physical and chemical properties parameters can be used as an illustration of the diversity of TALE proteins. The subcellular localization analysis of TALE protein was performed using the WoLF PSORT website (https://wolfpsort.hgc.jp/), which predicts the subcellular localization sites of proteins based on their amino acid sequences (Horton et al., 2007), and the protein secondary structures were predicted by using the online website SOMPA (https://npsa-prabi.ibcp.fr/cgi-bin/npsa_automat.pl?page=npsa%20_sopma.html) (Geourjon and Deleage, 1995).

### Localization, evolutionary relationships and covariance analysis of TALE family members

Download the gff annotated file from the Maize Genetics and Genomics Database (https://maizegdb.org/) (Woodhouse et al., 2021) and query the chromosome location of TALE family members. Based on the chromosomal position information and the types of structural domains contained, they were located on chromosomes by using TBtools (Chen et al., 2023). Genes containing POX and HD conserved domains were considered as BELL family, and genes with KNOX and HD conserved domains were considered as KNOX family. The segmentally and tandemly duplicated homologous of ZmTALE genes were examined to discover the potential biological implications of these patterns. The protein sequences of *Arabidopsis thaliana* TALE family were downloaded from TAIR (https://www.arabidopsis.org/) for reference. The phylogenetic tree was constructed using the neighbor-joining method (NJ, bootstrap = 1000) in MEGA7.0 software (Kumar et al., 2018). Obviously, TALE family can be divided into two subfamilies, KNOX and BELL. The intra-species and inter-species synteny analysis were performed by using the McscanX software (Wang et al., 2012). And the results were visualized by the Dual Systeny Plot for MCScanX program in the TBtools (Chen et al., 2023). The non-synonymous mutation frequency (Ka) and synonymous mutation frequency (Ks) of maize TALE family genes were analyzed using Advanced Ka/Ks Calculator in the TBtools (Chen et al., 2023).

### Analysis of codon usage pattern of ZmTALE genes

CodonW 1.4.4 program (J Peden, http://codonw.sourceforge.net) was used to analyze the usage pattern of ZmTALE genes, including nucleotide composition, GC content, ENC, the four types base in the third position of each codon, ect.

### Conserved motifs and gene structures analysis of ZmTALE genes

Motif analysis was performed using the online tool MEME (https://meme-suite.org/meme/tools/meme) (Bailey et al., 2009). Eight motifs were set to discover. Gene structures were visualized by using Gene Structure View program in TBtools (Chen et al., 2023).

### Analysis *cis*-elements of TALE gene family members in promoter

The 2000bp sequence upstream of ATG (promoter) of the maize TALE genes was obtained by using Gff3 Sequences Extract tool in TBtools (Chen et al., 2023). The PlantCARE online tool (http://bioinformatics.psb.ugent.be/webtools/) was used to predict the *cis*-elements in promoter regions (Lescot et al., 2002). Then visualized the cis-elements using EXCEL graphing.

### Expression patterns analysis of TALE genes in maize

The transcriptome dataset of maize genes was downloaded from the NCBI database GSE50191 (Walley et al., 2016), for tissues expression patterns analysis. And heat maps of the expression levels were plotted by using TBtools (Chen et al., 2023).

Total RNA was extracted using RNAiso Plus (TaKaRa, Japan). The concentration and purity of RNA was detected by NanoDrop2000 (Thermo). cDNA was obtained by reverse transcription reaction using Evo M-MLV RT Kit with gDNA Clean for qPCR (Accurate Biology). The cDNA was diluted for 30-fold for template. The genes specific primers were designed using Beacon Designer software (**Supplementary Table 1**). The expression levels of *ZmBELLs* and *ZmKNOXs* were detected by CFX96 PCR instrument (Bio-Rad, USA). The qRT-PCR reaction system consisted of 7.5 μL of 2× SYBR Green Pro Taq HS Premix (Accurate Biology), 0.3 μL upstream and downstream specific primers, 1.9 μL of ddH_2_O and 5 μL template. The reaction program was 95°C pre-denaturation for 30 sec; 95°C denaturation for 5 sec, 60°C for 30 sec, and 45 cycles. Melt curve 65°C to 95°C, increment 0.5°C. Three replications were performed for each sample, and the corresponding Ct values were obtained. The *Actin 1* was used for reference gene. The relative expression level was calculated by the 2^-ΔΔCt^ method (Schmittgen and Livak, 2008). Finally, the data is visualized using GraphPad prism.

### Subcellular localization analysis of *ZmKNOX05* and *ZmBELL11*

Specific primers (**Supplementary Table 1**) were designed to amplify the CDS sequence of the target genes without stop codon using fidelity DNA polymerase (Biorun, Wuhan). The PCR program is set as follows 94°C for 5 min, 30 cycles of 94°C for 30 sec, 50°C for 45 sec, 72°C for 30 sec, 72°C for 10min, 16°C for 30min. After electrophoresis, and gel DNA extraction, the target genes fragment was homologous recombinantly ligated to the linearized pBWA(V)HS-eGFP vector between CaMV35S promoter and enhanced green fluorescent protein tag using Biorun Seamless Cloning Kit (Biorun, Wuhan). The recombined vectors CaMV35S:TALE-eGFP or the empty vectors CaMV35S:eGFP, were transformed into the maize protoplasts according to the protocol (Yoo et al., 2007). The RFP vector containing a marker for nucleus localization, was co-transformed into protoplasts. After 18-24 hours of dark incubation, protoplasts were observed by laser confocal microscopy (Nikon, Japan).

## Results

### Identification, characterization and phylogenetic analysis of TALE family members in maize

**Figure 1:**
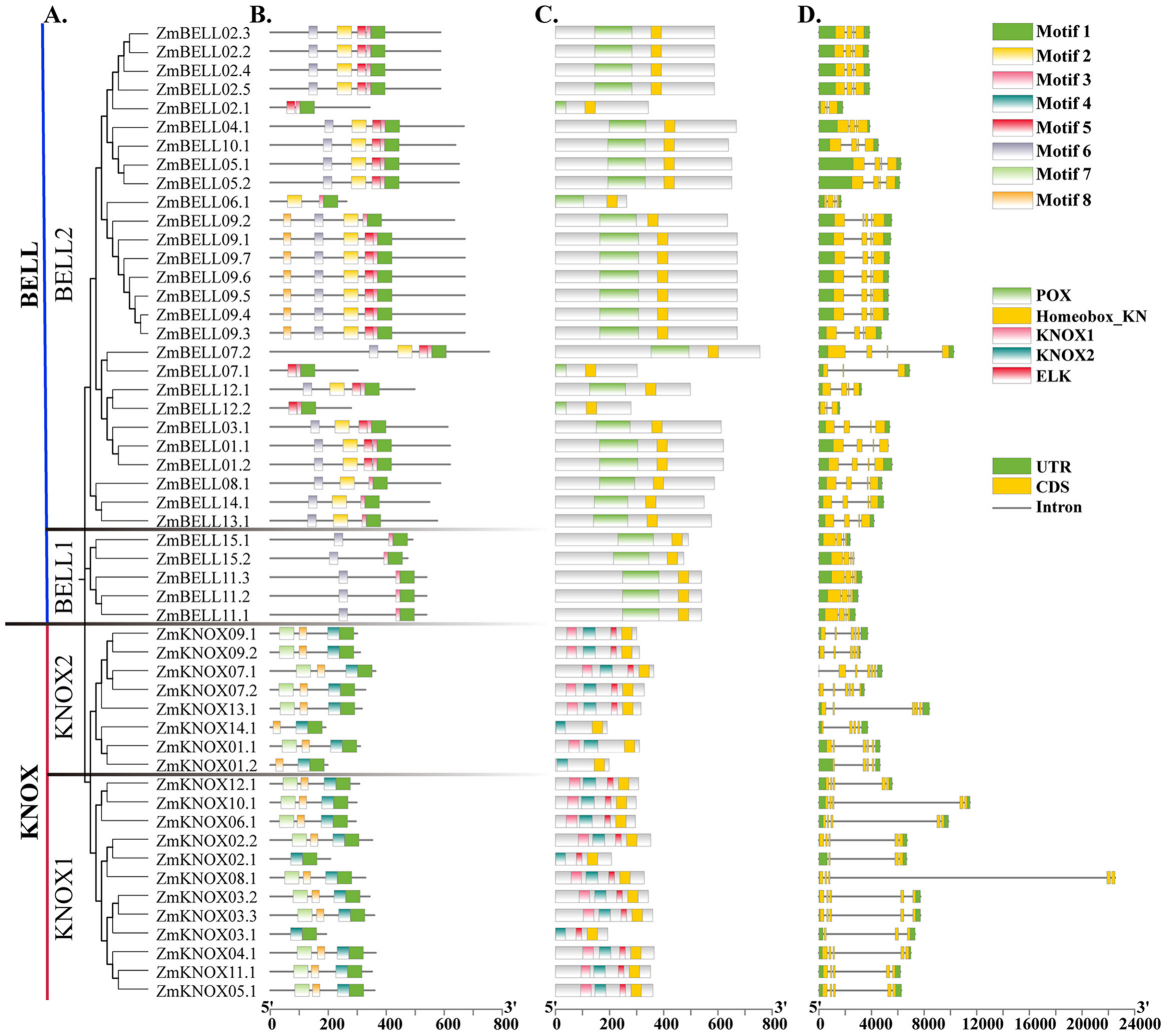
The classification, phylogenetic tree, conservative motifs and gene structures analysis of maize TALE family members. **(A)** A total of 52 TALE proteins were identified in maize, which were divided into two subfamilies, the KNOX and the BELL. KNOX were classified into KNOX1 and KNOX2, and BELL were classified into BELL1 and BELL2 subgroup. The unrooted neighbor-joining tree was constructed by using TALE protein sequences. **(B)** Motifs analysis of TALE proteins by MEME Suite. Different colored boxes represented different conserved motifs. **(C)** Conservative domains analyzed by NCBI-CDD. **(D)** Exon-intron structures of *ZmTALEs*. The exons were marked as yellow boxes, and the introns were represented by black lines; UTRs were shown as green boxes.

A total of 52 TALE proteins were identified in maize B73 RefGen_v3 genome. Obviously, they can be divided into two subfamilies, the KNOX and the BELL subfamily according to the domains they contained (**Figure 1A**). They all contained the homeobox domain (**Figure 1C**), which has three typical amino acids (PYP) between two helices (**Supplementary Figure 1**). In addition, most KNOX proteins contained KNOX1, KNOX2, and ELK domain, while 32 BELL proteins all contained POX domain. They were renamed on the chromosome according to their subfamily and location (**Supplementary Table 2**). Their molecular weight, protein length, and physical and chemical properties varied widely among subfamilies. The detailed characteristics were summarized in **Supplementary Table 2**. The KNOX subfamily genes length ranged from 3.145 kb to 22.510 kb, encoding proteins with an average length of 303.7 aa. The molecular weights ranged from 22.232 kDa (ZmKNOX14.1) to 40.276 kDa (ZmKNOX04.1), with an average of 33.594 kDa. The theoretical isoelectric point (pI) of these proteins ranged from 5.25 (ZmKNOX08.1) to 9.78 (ZmKNOX07.1), and 90% of the members (18/20) showed acidic properties. The KNOX proteins were all unstable proteins with an instability index greater than 40, with a grand average of hydropathicity (GRAVY) ranged from -0.934 (ZmKNOX03.1) to -0.458 (ZmKNOX07.2). The length of the BELL genes ranged from 1.575kb to 10.246 kb, and encoded proteins with an average length was 568.9 aa. The protein molecular weight of the BELL subfamily members ranged from 28.889 kDa (ZmBELL06.1) to 79.168 kDa (ZmBELL07.2), with an average of 60.5364 kDa. The pI values ranged from 5.22 (ZmBELL06.1) to 9.51 (ZmBELL12.2), with 81% (26/32) of the members exhibiting acidity. The BELL proteins instability index was greater than 40, similar to the KNOX proteins, indicating that they also belonged to unstable proteins. The GRAVY of BELL proteins ranged from -0.710 (ZmKNOX03.1) to -0.251 (ZmKNOX07.2). The above analysis indicated that members of the KNOX subfamily have longer gene lengths but smaller molecular weights and greater hydrophilicity than BELL subfamily members. Most members of both subfamilies are acidic and unstable proteins. Subcellular localization predicted by WoLF PSORT showed that they were mainly located on the nucleus (**Supplementary Table 2**).

The protein secondary structures were predicted using the SOMPA (https://npsa-prabi.ibcp.fr/cgi-bin/npsa_automat.pl?page=npsa_sopma.html). The results showed that the average percentage of α-helices and random coil were the highest, accounting for 43.37% and 45.64%, respectively (**Supplementary Table 2**). This may correlate with the homeobox domain contains three segments of α-helix. The high percentage of random coil may be related to the functional diversity of TALEs.

**Figure 2:**
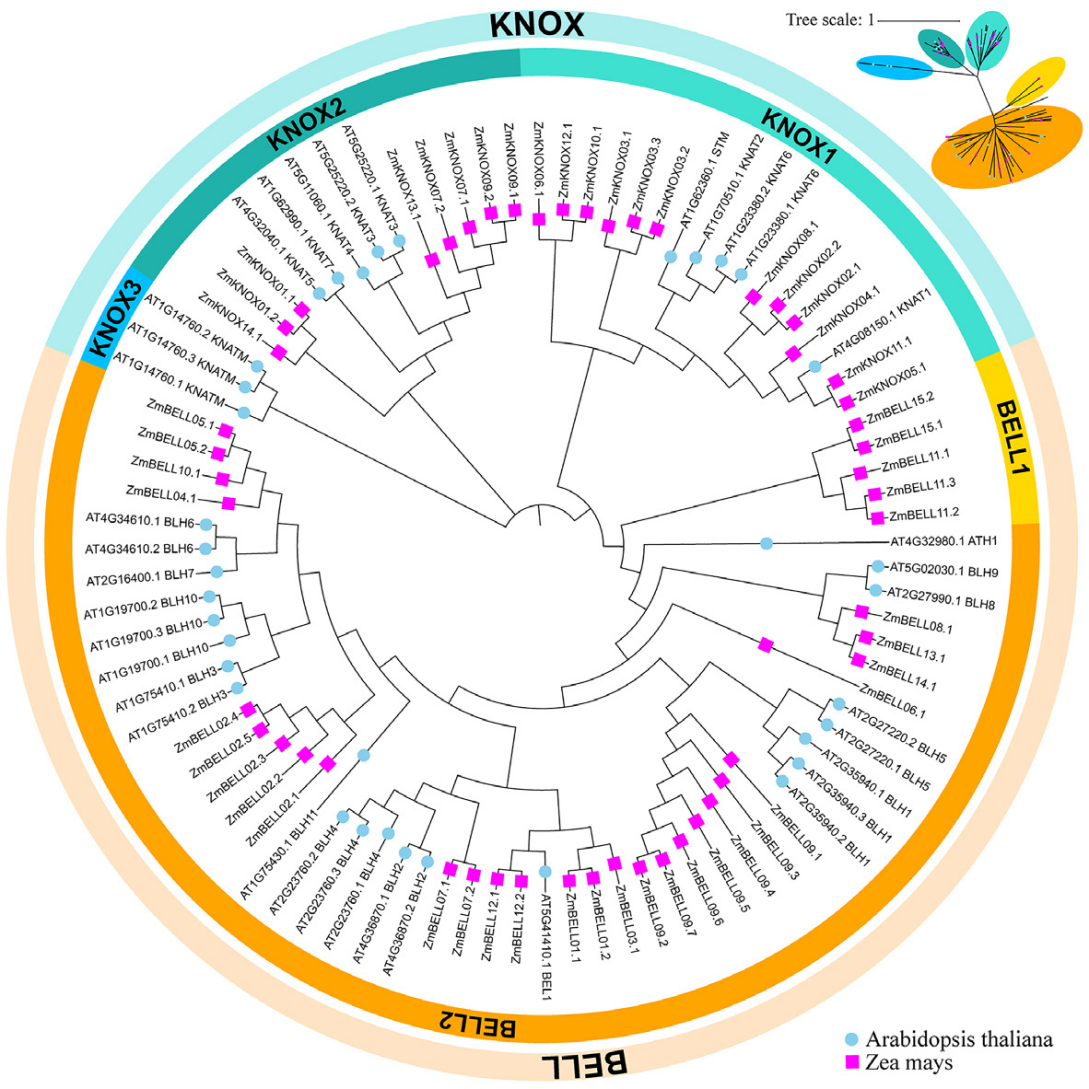
Evolutionary relationships of TALE transcription factors in maize and *Arabidopsis*. The phylogenetic tree was constructed by MEGA 6.0 software using the neighbor-joining (NJ) method. Bootstrap analysis was conducted with 1000 replicates. Zm indicates *Zea maize* (pink squares), At indicates *Arabidopsis thaliana* (blue dots). These TALE proteins were divided into two subfamilies based on their domains containing. On the periphery of the figure, the blue semi-circle represents the KNOX subfamily, and the orange semi-circle represents the BELL subfamily. The former can be further divided into KNOX1, KNOX2 and KNOX3 subclasses, and the latter into BELL1 and BELL2. Different branches were marked with different colors.

To investigate the phylogenetic and taxonomic relationships of the TALE family members, a phylogenetic tree was constructed using 88 TALE protein sequences, including 33 TALE members *from Arabidopsis thaliana* as references, and 52 TALE members from *zea mays* (**Figure 2**). We also constructed a phylogenetic tree only using TALE proteins from maize (**Figure 1A**). Similar to *Arabidopsis thaliana*, the TALE family in maize was clearly divided into two well-conserved subfamily, KNOX and BELL. TALE proteins in maize were further divided into four classes KNOX1, KNOX2, BELL1 and BELL2, and did not contain the dicot-specific KNOX3 proteins (KNATM). Among the four classes, BELL2 members were the most numerous, accounting for about 51.9% (27/52). BELL1 class contained only five maize TALE members and no Arabidopsis TALE family members, suggested that the maize TALE family has expanded in the BELL class over a long evolutionary period.

### Chromosome distributions of TALE genes

**Figure 3:**
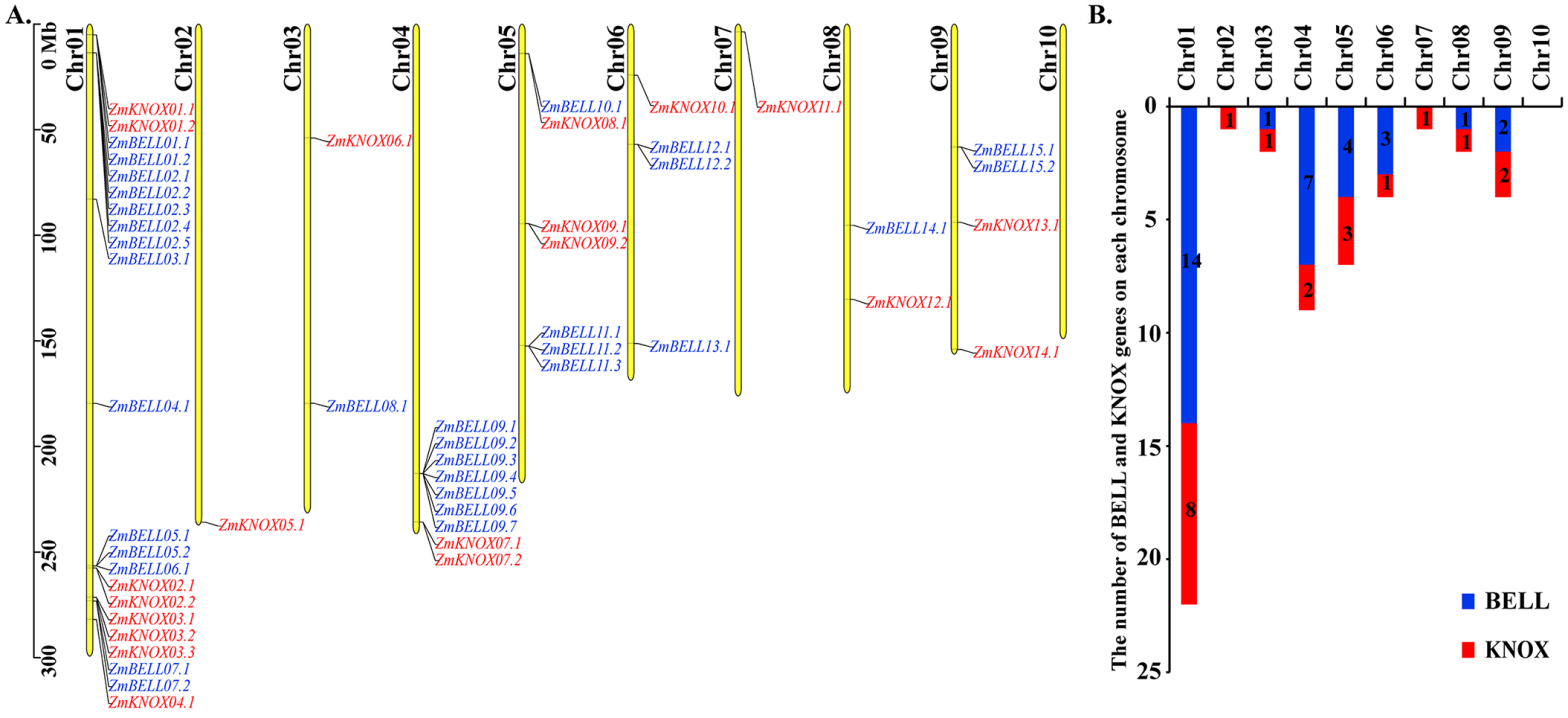
Location and number of maize *TALEs* in chromosome. **(A)** The distribution and location of the maize *TALEs* on the chromosome, where the blue indicates the BELL subfamily genes and the red indicates the KNOX subfamily genes. Chromosome numbers are on the left and *ZmTALEs* are on the right of chromosomes. The scale is in mega bases (Mb). **(B)** The number of ZmKNOX and ZmBELL genes on each chromosome.

All 52 TALE transcripts were located on chromosomes Chr01 to Chr09, with no distribution on Chr10 (**Figure 3A**). These members were unevenly distributed throughout the genome. Among them, Chr01 has the largest number of TALE genes, accounting for 42.3% (22/52), including 14 members of the BELL subfamily and 8 members of the KNOX subfamily, while 17.3% (9/52) of the members were distributed on Chr04. Chr01, Chr04 and Chr05 contained most TALE members (36/52), and only 7 ZmBELL and 7 KNOX genes were distributed on the rest of the chromosomes (**Figure 3B**). ZmTALE genes on chromosomes 1, 2, 4, and 7 were predominantly located at the distal ends. Among all ZmTALE genes, the distance between ZmBELL05 and ZmBELL06 was less than 200kb, which was a tandem duplicate gene (Elemento et al., 2002).

### Collinearity analysis of TALE genes in maize

**Figure 4:**
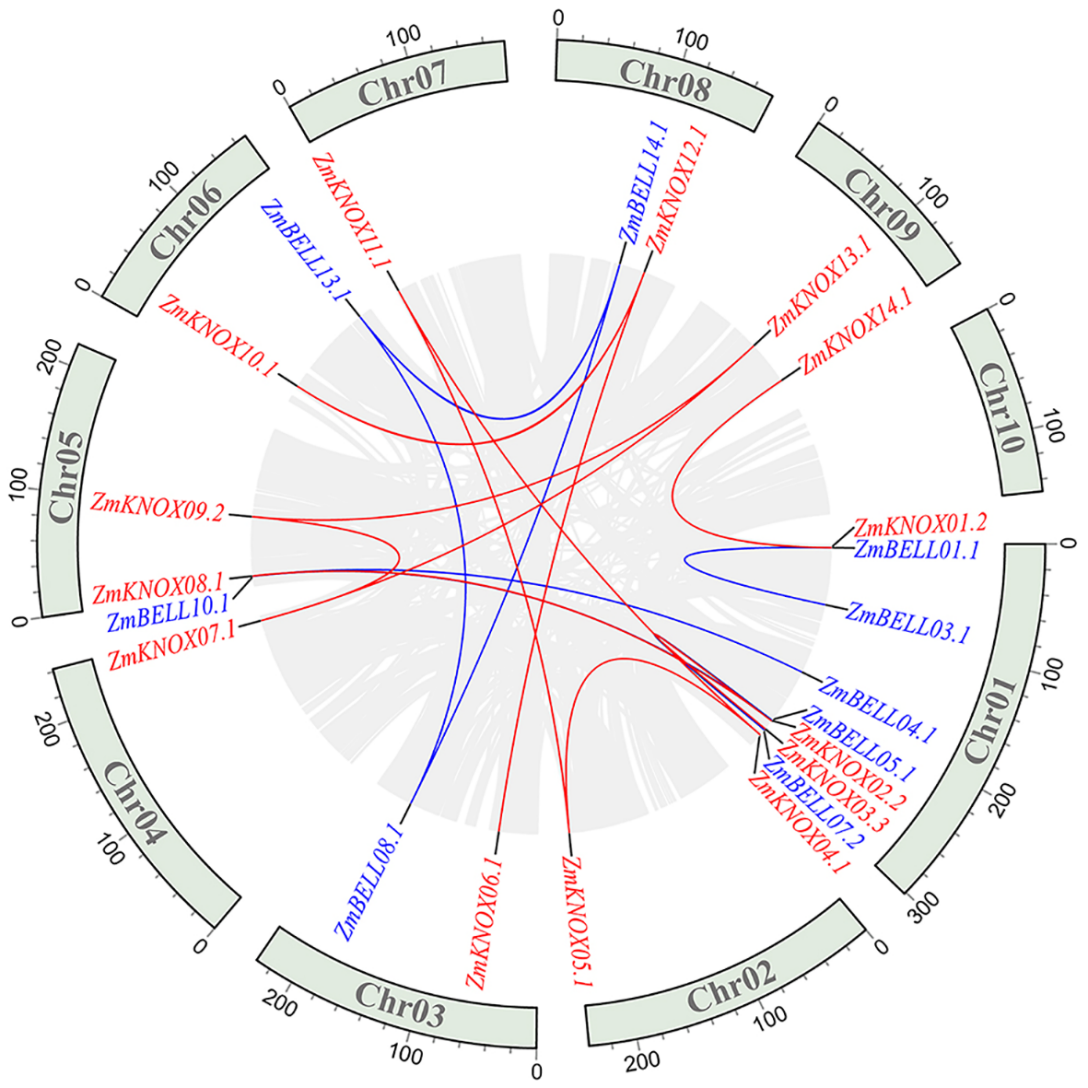
Synteny analysis of *ZmTALEs* in maize genome. The blue indicates the BELL subfamily genes and the red indicates the KNOX subfamily genes. Red lines represent collinear gene pairs between KNOX genes, blue lines represent collinear gene pairs between BELL family genes, and grey lines represent other collinear gene pairs of non-TALE gene members within the genome. The collinearity relationship of different gene pairs was performed using MCScanX software.

In order to investigate the expansion of TALE members in maize, we performed intra-species collinearity analysis. A total of eighteen collinear pairs involving 23 TALE genes were found, including14 BELL members and 9 KNOX members (**Figure 4**). There are three collinear gene pairs on Chr01 (ZmBELL01.1 and ZmBELL03.1, ZmBELL05.1 and ZmBELL07.2, ZmKNOX02.2 and ZmKNOX03.3), which were further indicated in yellow in **Supplementary Table 3**. Compared to the tandem duplications, the segmental duplication events mainly drove the expansion of ZmTALE superfamily. The non-synonymous mutation frequency (Ka) and synonymous mutation frequency (Ks) of maize TALE genes were analyzed using Calculator software, and the Ka/Ks of gene pairs were calculated. The result showed the Ka/Ks values of all pairs of genes were less than 1 (**Supplementary Table 3**), indicating that maize TALE genes were subjected to a robust purify selection pressure to cope with hazards, such as biotic and abiotic stresses in nature during evolution.

**Figure 5:**
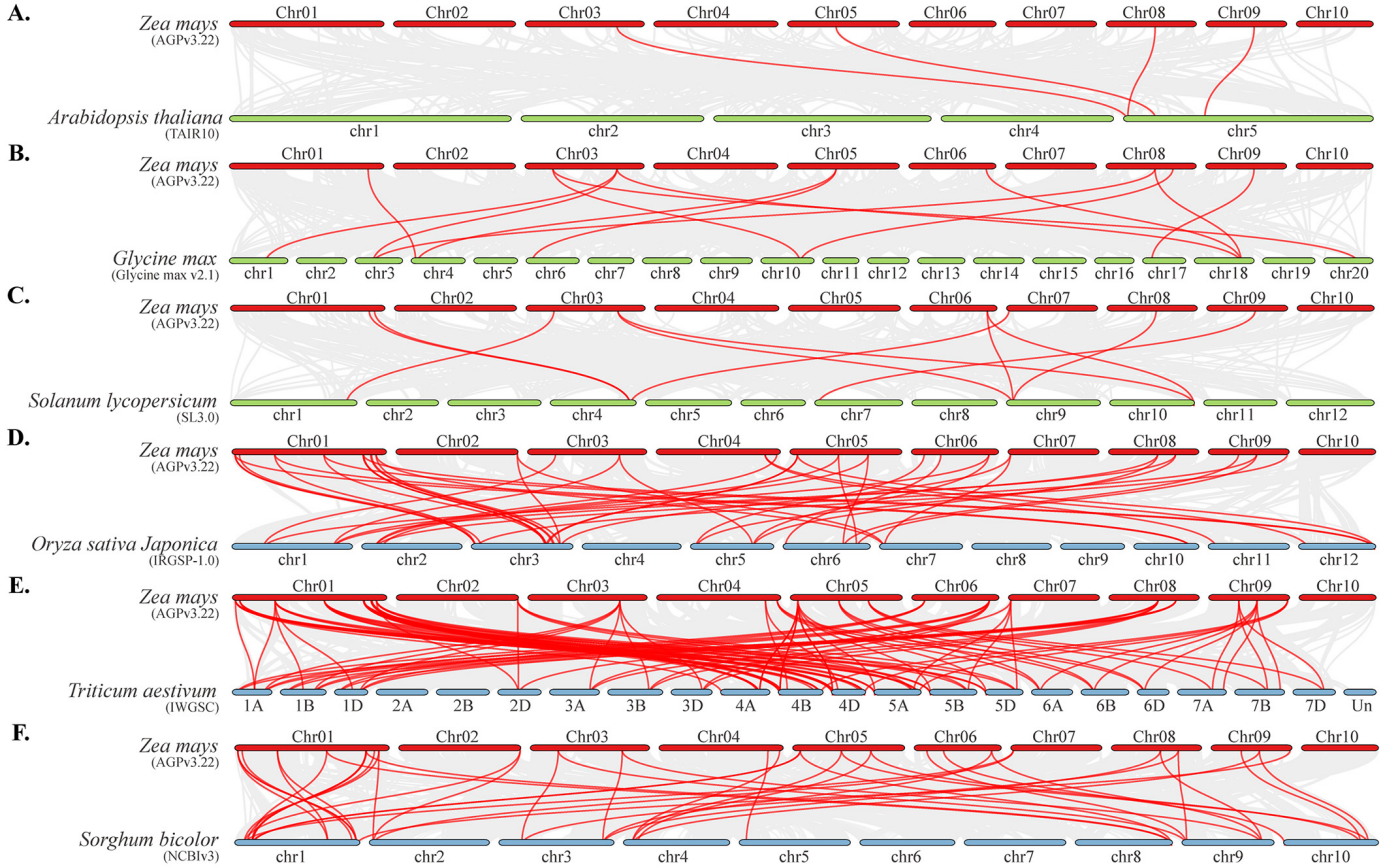
The collinearity analysis of the ZmTALE family members between *zea mays* and and six representative plant species. **(A–C)** represent gene pairs with a collinear relationship between maize and three dicotyledons, including *Arabidopsis thaliana*, *Glycine max*, and *Solanum lycopersicum*, respectively. **(D–F)** represent the collinearity analysis of gene pairs between maize and three monocotyledons, including *Oryza sativa Japonica*, *Triticum aestivum*, and *Sorghum bicolor*, respectively. Gray lines in the background indicated the collinear blocks within maize and other plant genomes, and the red lines highlighted the colinear ZmTALE gene pairs.

To further investigate the evolutionary clues of TALE family between maize and other species, we performed collinearity analyses between maize and monocotyledons and dicotyledons. The co-linear gene pairs with dicotyledons (*Glycine max*, S*olanum lycopersicum*, *Arabidopsis thaliana*) were mainly present on chromosomes 3, 8, and 9, whereas the co-linear gene pairs with monocotyledons (*Sorghum bicolor*, *Oryza sativa*, and *Triticum aestivum*) were distributed on each chromosome (except chromosome 10) (**Figure 5**). Obviously, the number of co-linear gene pairs of TALE genes between maize and the three monocotyledonous plants was significantly higher than that of the three dicotyledonous. To confirm this hypothesis, we increased the number of species of monocotyledons and dicotyledons (**Supplementary Table 3**). The results showed monocotyledon plants and maize have collinear gene pairs ranging from 41 to 105, with an average of 55, involving 29 maize TALE genes. The number of collinear gene pairs between dicotyledonous plants and maize ranges from 4 to 14, with an average of 7, involving 11 maize TALE genes (**Supplementary Figures 2A, B**). Moreover, these 11 TALE genes also had collinearity within maize genome (**Supplementary Figure 2C**). These results indicated that the TALE family genes of maize were more homologous to monocotyledons than to dicotyledons. Some TALE genes (such as ZmBELL08.1 and ZmBELL14.1) may already exist before the differentiation of monocotyledonous and dicotyledonous plants, and some TALE genes were generated through the evolution and expansion of monocotyledonous plants. Combined with the results of intra-species collinearity analyses, some TALE genes were extended and evolved within maize species, even on the same chromosome.

### Analysis of codon usage pattern of maize TALE genes

**Figure 6:**
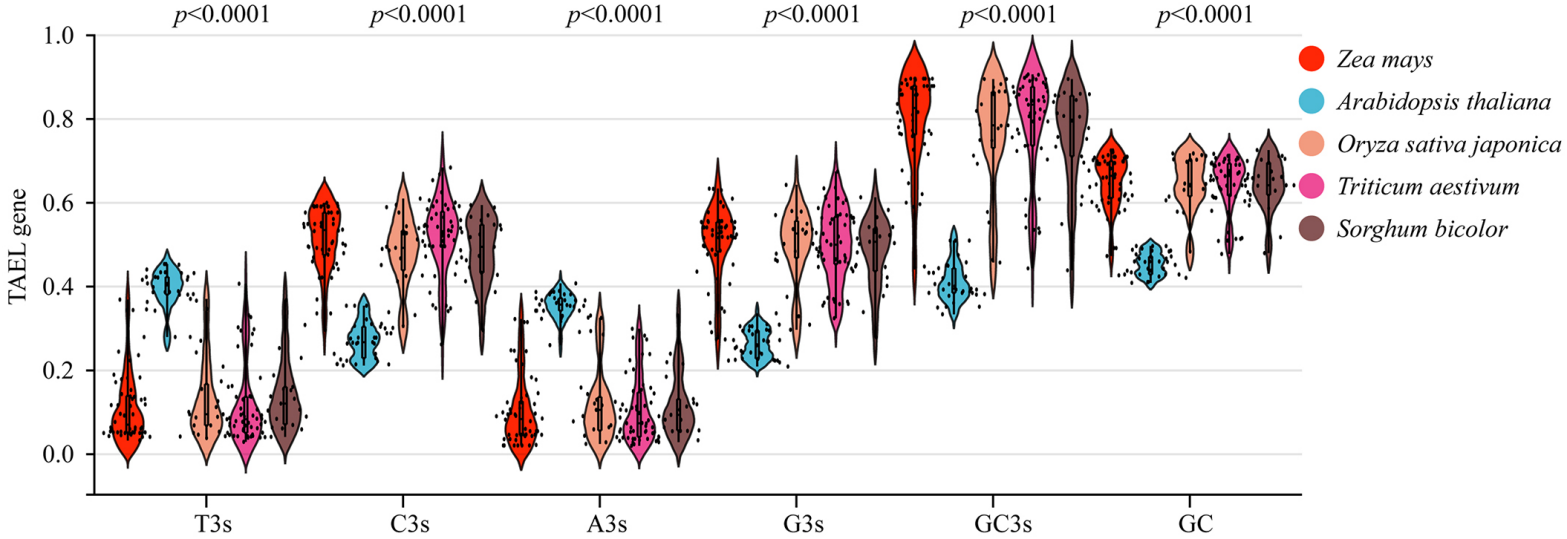
Violin plot of T3s, C3s, A3s, G3s, GC3s, and GC contents in *zea mays*, *Arabidopsis thaliana*, *Sorghum bicolor*, *Oryza sativa*, and *Triticum aestivum* of TALE genes. T3s, C3s, A3s, G3s represented the frequency of the third corresponding base (T, C, A, G) of all codons in the gene. GC3s referred to the frequency at which G and C appear in the third position of the codon. GC contents referred to the 3rd GC content of all codons in the gene (except methionine, tryptophan, and stop codon). Red for maize, blue for Arabidopsis, orange for rice, pink for wheat, and brown for sorghum.

Codons play essential roles in the transmission of biological genetic information. The codon usage patterns are thought to be related to the GC content of the third codon location (GC3) (Mazumdar et al., 2017). We analyzed the codon usage patterns of TALE genes using the CDS sequences from five species (*zea mays*, *oryza sativa* subsp. japonica, *triticum aestivum*, and *Arabidopsis thaliana*) (**Supplementary Table 4**). We observed that the average C3s, G3s, GC3s and GC contents of TALE genes were higher in monocots than in dicots (Arabidopsis thaliana), and the p values were highly significant (**Figure 6**). Effective number of codons (ENC), codon adaptation index (CAI), codon bias index (CBI), and frequency of optimal codons (Fop), were used to assess the codon usage bias indices (Parvathy et al., 2022). The ENC of *Arabidopsis thaliana* (dicotyledonous plant) was higher than that of monocotyledonous plants (maize, rice, sorghum, wheat) (**Supplementary Table 4**). Among several species, maize had the lowest average ENC (40.125) and the highest average CAI (0.263), CBI (0.216) (**Supplementary Table 4**). This indicated that maize had the strongest adaptability, while *Arabidopsis thaliana* had the weakest adaptability among the five crops (CAI=0.210). This may be related to the evolutionary relationship of the species.

### Conserved motifs and structures of maize TALE genes

To gain a deeper understanding of the functional diversity of TALE genes, we used MEME (http://meme-suite.org/tools/meme) to predict conserved motifs of maize TALE proteins. Eight conserved motifs were analyzed. The sequences of conserved motifs were shown in **Supplementary Figure 3**. The results indicated that the motifs of proteins in the same subfamily were almost identical in composition and order of arrangement (**Figures 1A, B**). Most of the KNOX subfamily contained four motifs, Motif 1, Motif 4, Motif 7, and Motif 8, which was roughly consist with HD, ELK, KNOX1 and KNOX2 domain, respectively. HD consists of 60 amino acids, which can form three helical structures. ELK acts as nuclear localization signal. KNOX1 and KNOX2 domains have been reported to form the MEINOX structural domain, which may function in heterodimers of KNOX and BEL1-like proteins interactions (Burglin, 1997). The BELL subfamily all contained Motif 1, Motif 3 and Motif 6. BELL1 subclass contained only these three motifs, while BELL2 subclass also contained Motif 2, Motif 5 and Motif 8. These motifs were associated with the HD and POX domains. The POX domain can interact with the MEINOX to form dimer to regulate plant growth and development, and response to stress (Niu and Fu, 2022). Motif 1 partially overlaps with homeobox domain, and all contain “PYP”, a sequence shared by members of the TALE family (**Supplementary Figure 3**). Differences in protein motifs among subfamilies may elucidate the functional diversity of KNOX and BELL proteins. For example, the domains KNOX2 and HD of the rice KNOX protein OSH15, are essential for inducing abnormal phenotype, while the KNOX1 and ELK domains influence phenotypic severity by inhibiting gene expression (Niu et al., 2022). In general, proteins with similar motifs tend to cluster together in phylogenetic analyses, implying that members of the same sub-branch have similar functions, and that members clustered in different branch may function differently.

To investigate the gene structure of *ZmTALEs*, we visualized the.gff file of the maize TALE genes using TBtools. We found that the maize TALE genes all had 3’-UTR or 5’-UTR. The number of introns in TALE genes ranged from 2 to 6, and the number of exons ranged from 3 to 7. Most members of the KNOX1 subclass contained long introns, especially *ZmKNOX08.1* (**Figure 1D**). In general, BELL subfamily genes contained shorter introns than those of the KNOX subfamily members. These results indicated that there were some similarities in gene structure within subfamilies and differences in gene structure among different subfamilies.

### *Cis*-elements analysis of maize TALE genes promoter

**Figure 7:**
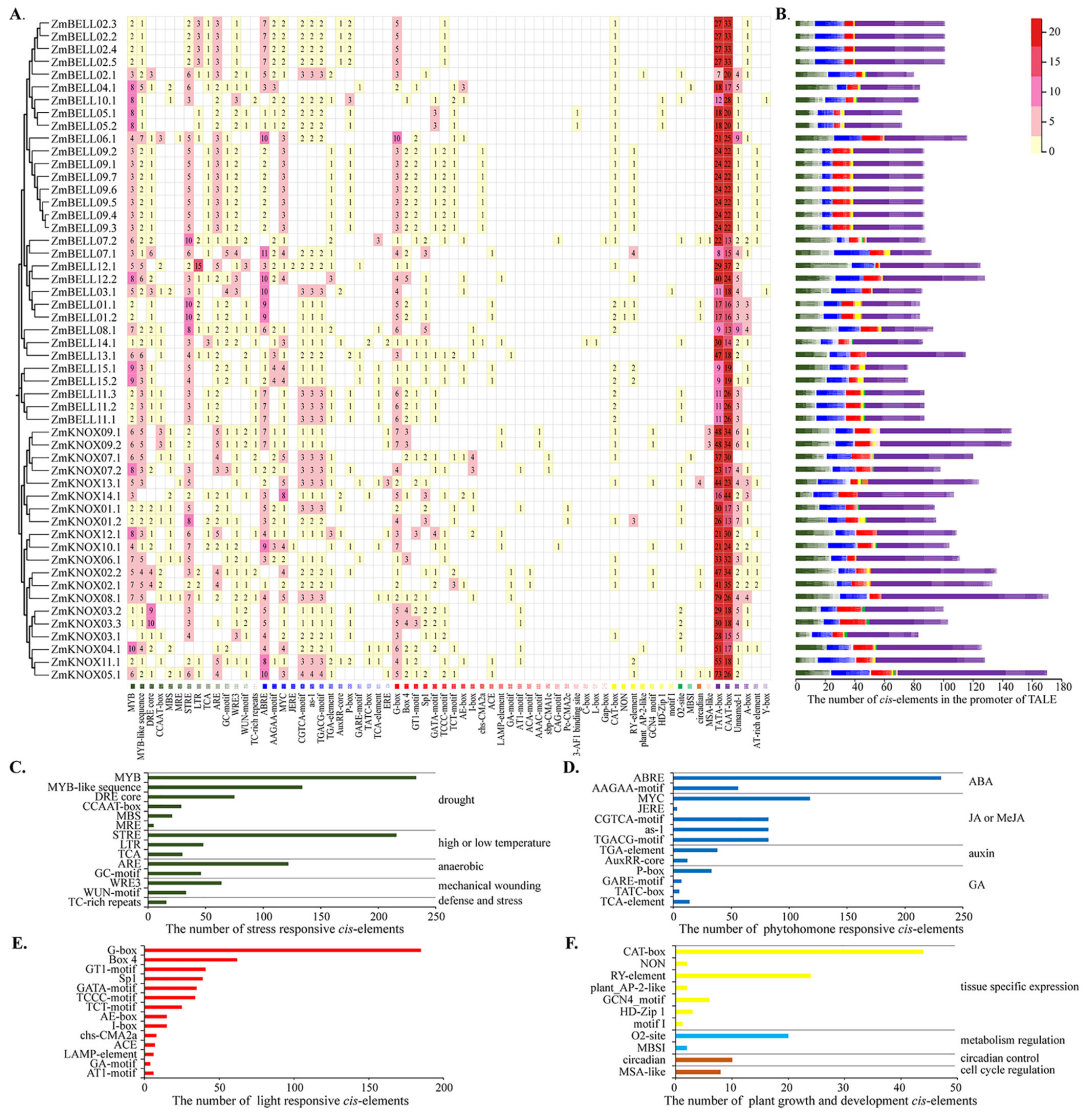
Analysis of *cis*-elements in the promoter of maize TALE genes. **(A)** Heat map of the number of *cis*-elements contained in the promoter region of each *ZmTALE* gene. Numbers represented the number of *cis*-elements, white indicated the absence of the *cis*-element. Different colored boxes represented different *cis*-elements. **(B)** Summary plots of the number of different types and number of *cis*-elements in **(A)**. Types and number of *cis*-elements related to stress-related **(C)**, hormone-responsive **(D)**, light-responsive **(E)**, and related to growth and development **(F)**, in the promoter regions of *ZmTALEs*.

*Cis*-elements in the promoter play crucial roles in the regulation of gene expression. To further understand the potential regulatory and expression mechanisms of TALE genes, we predicted *cis*-elements in the promoter of each gene using the PlantCARE database (http://bioinformatics.psb.ugent.be/webtools/) (**Supplementary Table 5**). **Figures 7A, B** illustrate the distribution and number of core promoter *cis*-elements, respectively. An average of 102 *cis*-elements were identified in the promoter region of each gene, and the most abundant of which were core promoter elements such as TATA-box and CAAT-box (**Figures 7A, B**). Among them, we selected elements associated with abiotic and biotic stress-responsive, phyhormone-responsive, light-responsive, and plant growth and development for analysis and statistics. Most of the stress responses *cis*-elements were related to drought response (MYB, MYB-like sequence, DRE core, CCAAT-box, MBS, MRE), followed by stress response element (STRE). There were also anaerobic (ARE), wounding and defensive responses (such as WRE3, WUN-motif, TC-rich repeats). Surprisingly, the number of MYB elements in response to drought stress and STRE elements in response to stress had exceeded 200, and they were distributed on the promoters of almost each TALE gene (**Figures 7A, C**). The hormone response elements were mainly related to abscisic acid (ABRE, AAGAA-motif), followed by jasmonic acid (MYC, CGTCA-motif, as-1, TGACG-motif, etc.) (**Figure 7D**). The number of light-responsive elements, and plant growth and development-responsive elements were also very high (**Figures 7E, F**). Because MYB, STRE, and ABRE *cis*-elements have been reported to be related to abiotic stress and hormonal regulation process (Liu et al., 2014; Ebeed, 2022), so we infer that the TALE genes in maize may be involved in the response to abiotic stress and phyhormone.

### Expression profiling of *ZmTALEs* in different tissues

**Figure 8:**
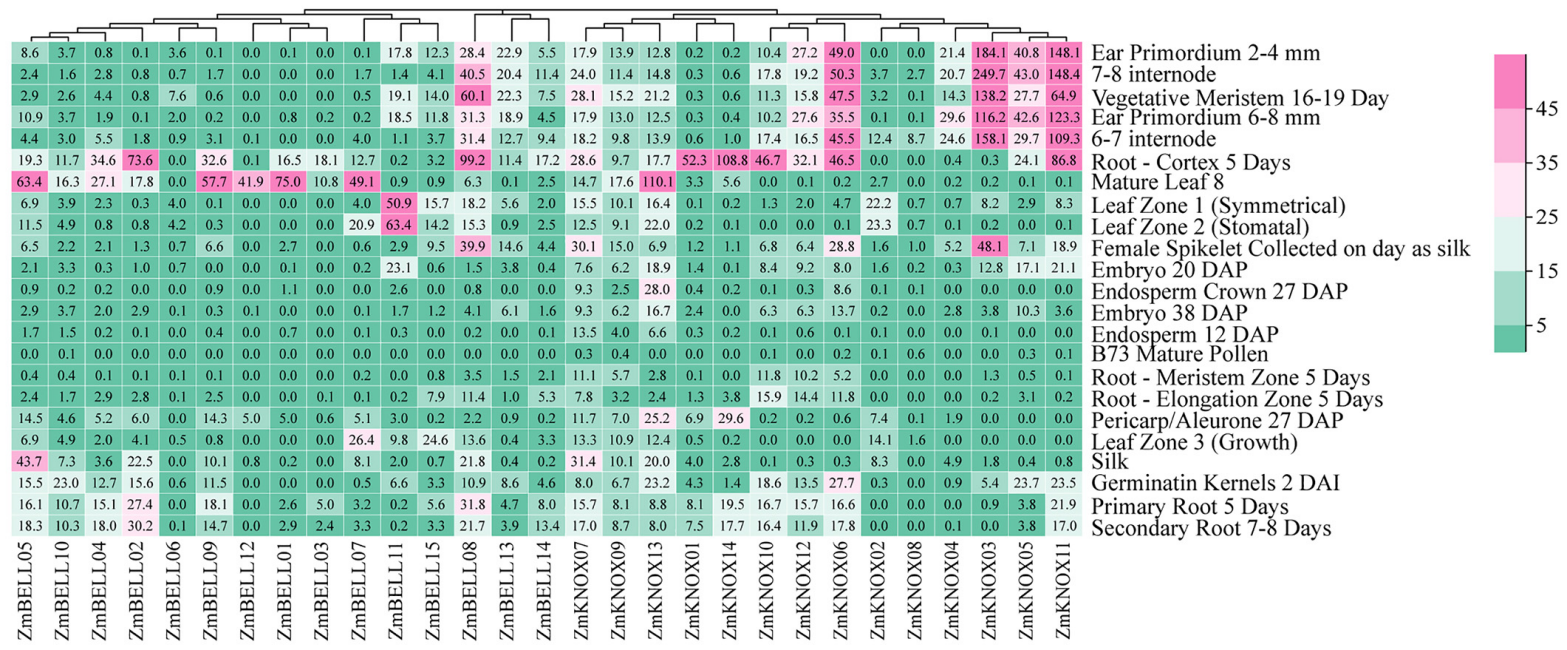
The expression profiles of *ZmTALE* genes in different tissues, and growth and development stages based on RNA sequencing data. The numbers represent FPKM values.

Spatial-temporal patterns of gene expression can often provide valuable clues on gene function. To understand the expression profile of TALE genes in different tissues and growth stages of maize, we downloaded maize B73 transcriptome data from the published RNA-seq data (Walley et al., 2016). The tissue-specific expression heat map was drawn as shown in **Figure 8**. We found that 52.8% (352/667) of TALE genes showed very low or no expression (FPKM<3) in 23 tissue sites, with 58.6% of the BELL subfamily genes (202/345), and 46.6% of the KNOX subfamily genes (150/322) (**Supplementary Table 6**). There were 4 genes highly expressed (FPKM>15) in more than 10 tissues, including one BELL subfamily gene (*ZmBELL08*, 12 tissues) and three KNOX subfamily genes (*ZmKNOX07*, *ZmKNOX11*, and *ZmKNOX13*, 11 tissues) (**Supplementary Table 6**). The expression levels of TALE genes were relatively low in endosperm and pollen. The KNOX1 subfamily genes (especially *ZmKNOX06*, *ZmKNOX03*, *ZmKNOX05*, *ZmKNOX11*) were highly expressed in ear primordium (2-4 mm, 6-8 mm), internode (6-7, 7-8), vegetative meristem, and root-cortex. That is, KNOX1 members were highly expressed in the primordium, meristem and root, where growth was vigorous. And KNOX2 members were highly expressed in roots. However, the BELL family members were highly expressed in leaves. The different tissue expression patterns indicated that they have different functions in plant growth and development.

### Expression patterns of *ZmTALEs* under ABA hormone, drought, salt, and heat stresses

**Figure 9:**
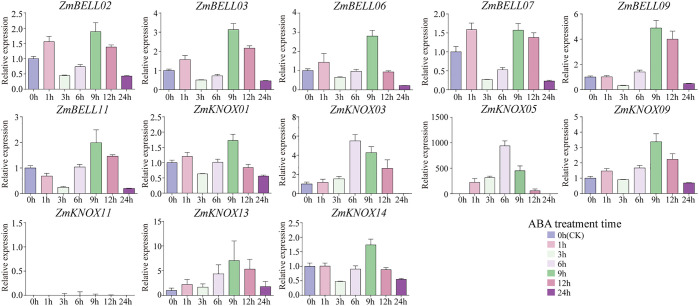
The expression levels of *ZmTALEs* under ABA hormone treatment. The three-leaf stage seedlings were treated with 100 µM ABA, and the expression levels of *ZmTALEs* were detected by RT-qPCR. Seedlings with no treatment as control. The bars indicate the mean ± SD of three replicates. The X-axis indicated different time points after ABA spray.

Previous studies have shown that TALE genes are involved in plant response to high temperature, drought, salinity and hormone stress (Zhao et al., 2019; Peng et al., 2021; Wang et al., 2021; Han et al., 2022; Rathour et al., 2022; Yang et al., 2022; Liao et al., 2024). To investigate the expression patterns of the TALE genes in maize under abiotic stress, we selected 13 genes, including six from the BELL family (one from the BELL1 family and five from the BELL2 family) and seven from the KNOX family (three from the KNOX1 family and four from the KNOX2 family), and examined their expression levels by qRT-PCR after ABA, NaCl, PEG-6000, and high temperature stress treatments, respectively (**Supplementary Table 7**). The results showed that after ABA hormone treatment 1-h, 3-h, 6-h, 9-h, 12-h, and 24-h, the relative expression of most *ZmTALEs* showed a tendency to increase and then decrease, and then increase, and then decrease, except *ZmBELL11*, *ZmKNOX03*, *ZmKNOX05*, and *ZmKNOX11* (**Figure 9**). The expression of most *ZmTALEs* increased after 1-h of treatment, decreased after 3-h, increased after 6-h, rose to the highest after 9-h, and then started to decrease. The expression of *ZmBELL11* showed a trend of down-regulation first, then up-regulation, and then down-regulation. *ZmKNOX03*, *ZmKNOX05*, and *ZmKNOX11*, which belong to KNOX1 family, all showed a trend of first up-regulation and then down-regulation, and its expression increased to the highest level after 6-h, and then decreased (**Figure 9**).

**Figure 10:**
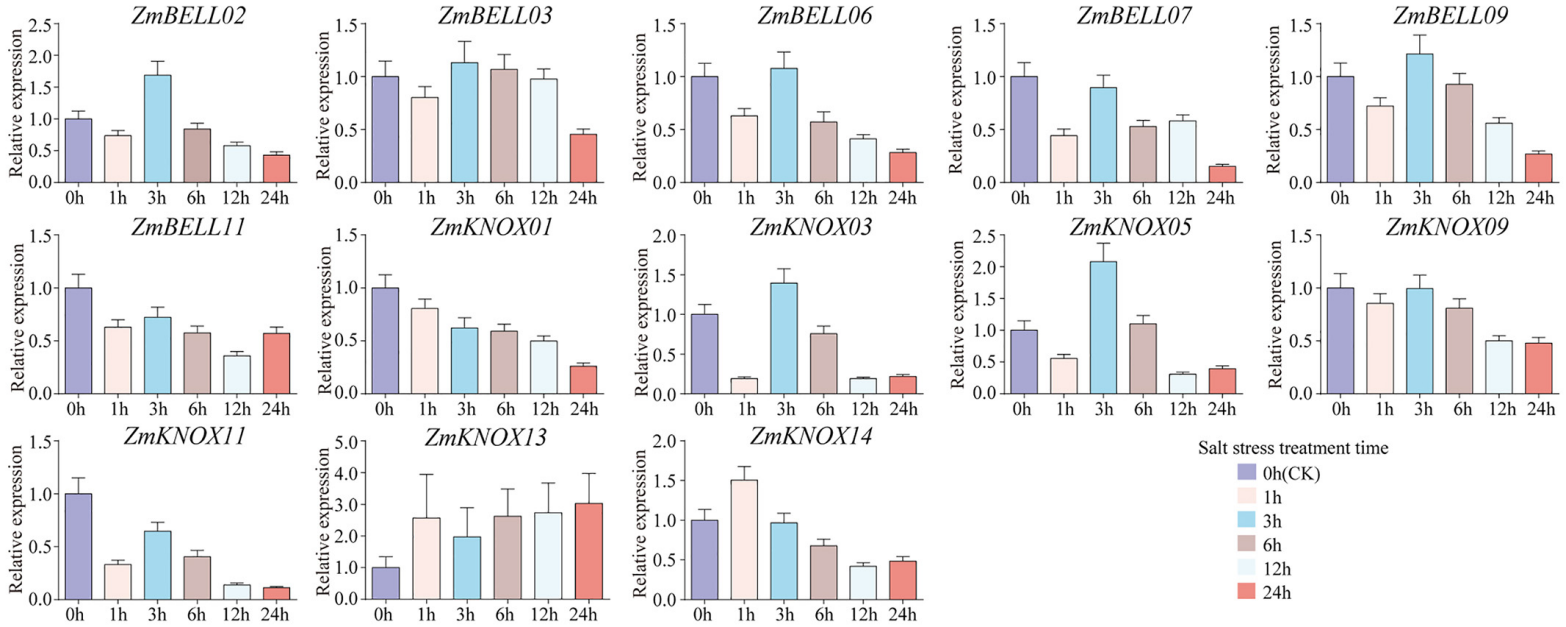
The expression levels of *ZmTALEs* under salt stress treatment. The three-leaf stage seedlings were treated with 200mM NaCl, and the expression levels of *ZmTALEs* were detected by RT-qPCR. Seedlings with no treatment as control. The bars indicate the mean ± SD of three replicates. The X-axis indicated different time points after NaCl watered.

**Figure 11:**
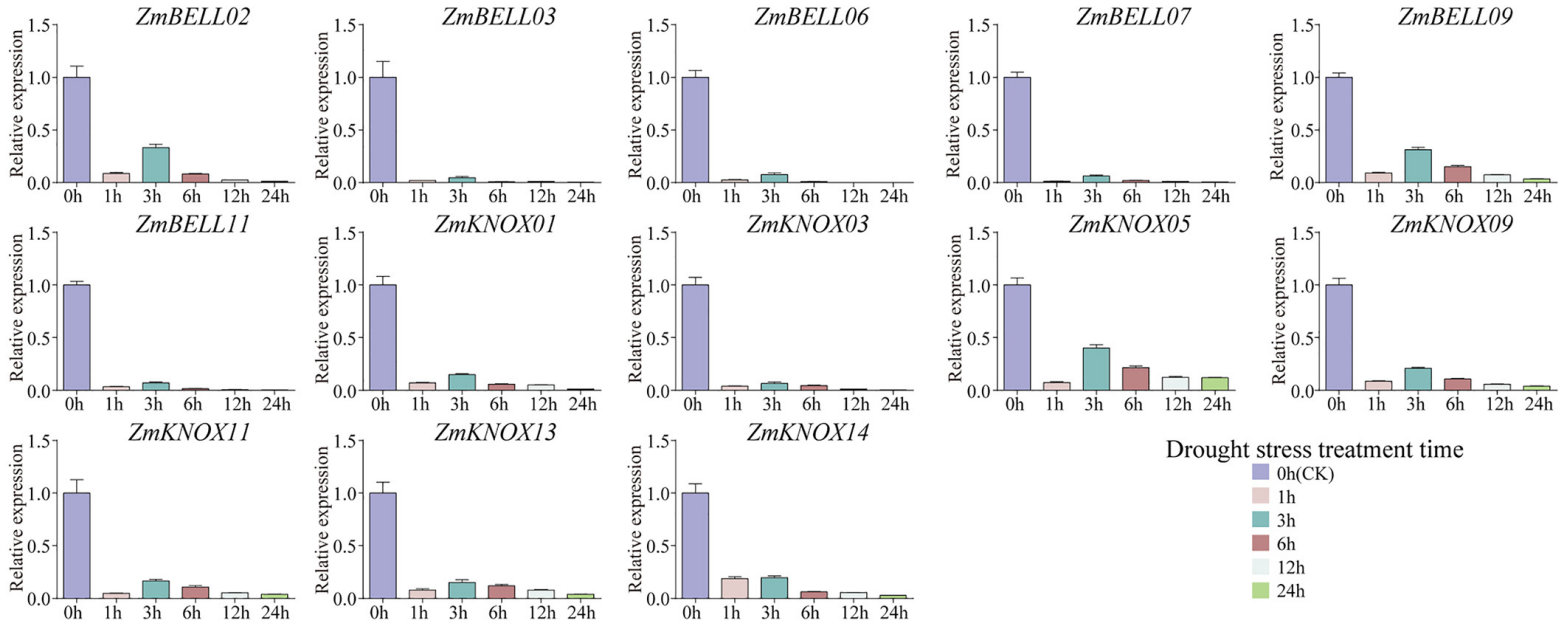
The expression levels of *ZmTALEs* under drought treatment. The three-leaf stage seedlings were treated with 35% PEG-6000 (w/v) solution, and the expression levels of *ZmTALEs* were detected by RT-qPCR. Seedlings with no treatment as control. The bars indicate the mean ± SD of three replicates. The X-axis indicated different time points after PEG-6000 watered.

**Figure 12:**
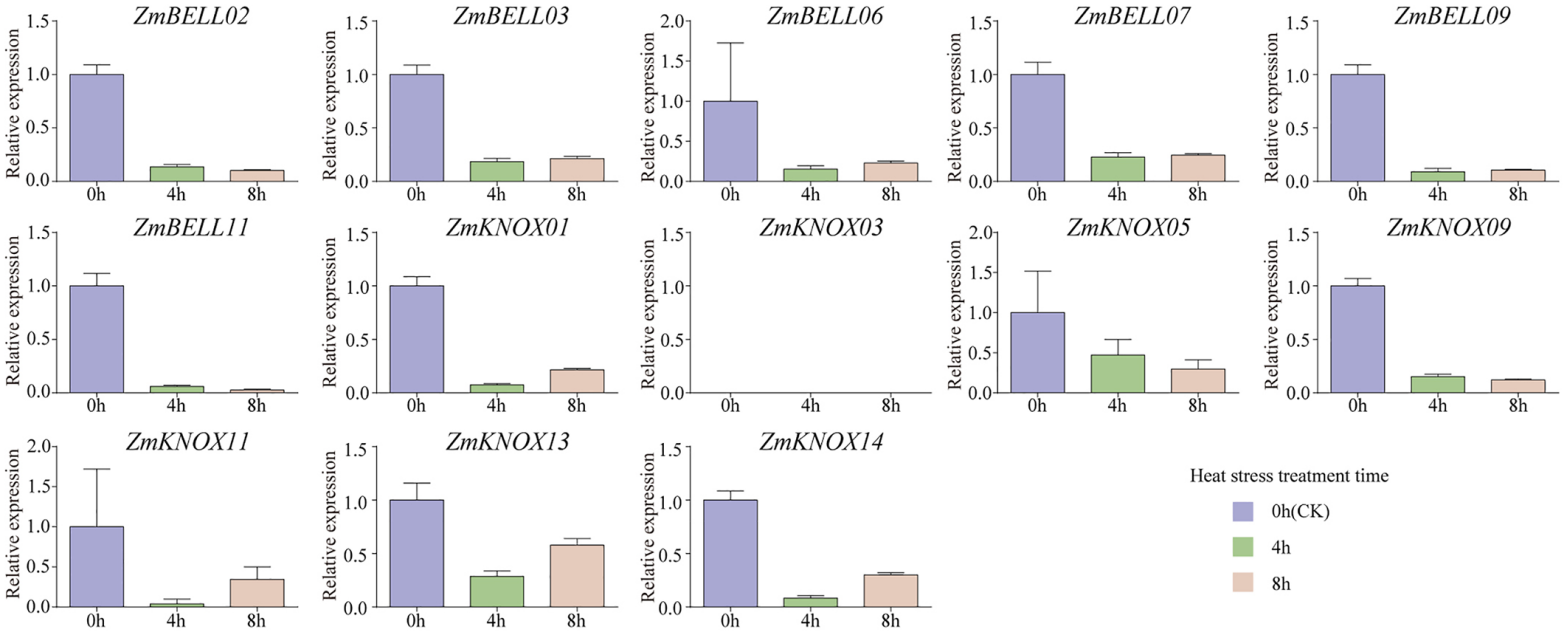
The expression levels of *ZmTALEs* in leaf under heat treatment. The three-leaf stage seedlings were treated with 42°C, and the expression levels of *ZmTALEs* were detected by RT-qPCR. Seedlings with no treatment as control. The bars indicate the mean ± SD of three replicates. The X-axis indicated different time points after heat treatment.

The expression levels of most *ZmTALEs* (such as *ZmBELL02*, *ZmBELL03*, *ZmBELL06*, *ZmBELL07*, *ZmBELL09*, *ZmKNOX03*, *ZmKNOX05*, *ZmKNOX09*, and *ZmKNOX11*) showed a trend of decreasing, then increasing and then decreasing after salt stress. The expression of *ZmBELL11* showed a trend of decreasing, and then increasing. The expression of *ZmKNOX01* showed a decreasing trend, however, *ZmKNOX13* showed an increasing trend. *ZmKNOX14*, on the other hand, showed a trend of increasing and then decreasing (**Figure 10**). In addition, the expression of all *ZmBELLs* and *ZmKNOXs* detected decreased significantly after PEG-6000 simulated drought stress and heat stress (**Figures 11**, **12**). These studies indicated that TALE transcription factors may negatively regulate maize plant growth and development under stress conditions such as drought, heat, high salt, and ABA hormone.

### Subcellular localization of *ZmKNOX05* and *ZmBELL11*

**Figure 13:**
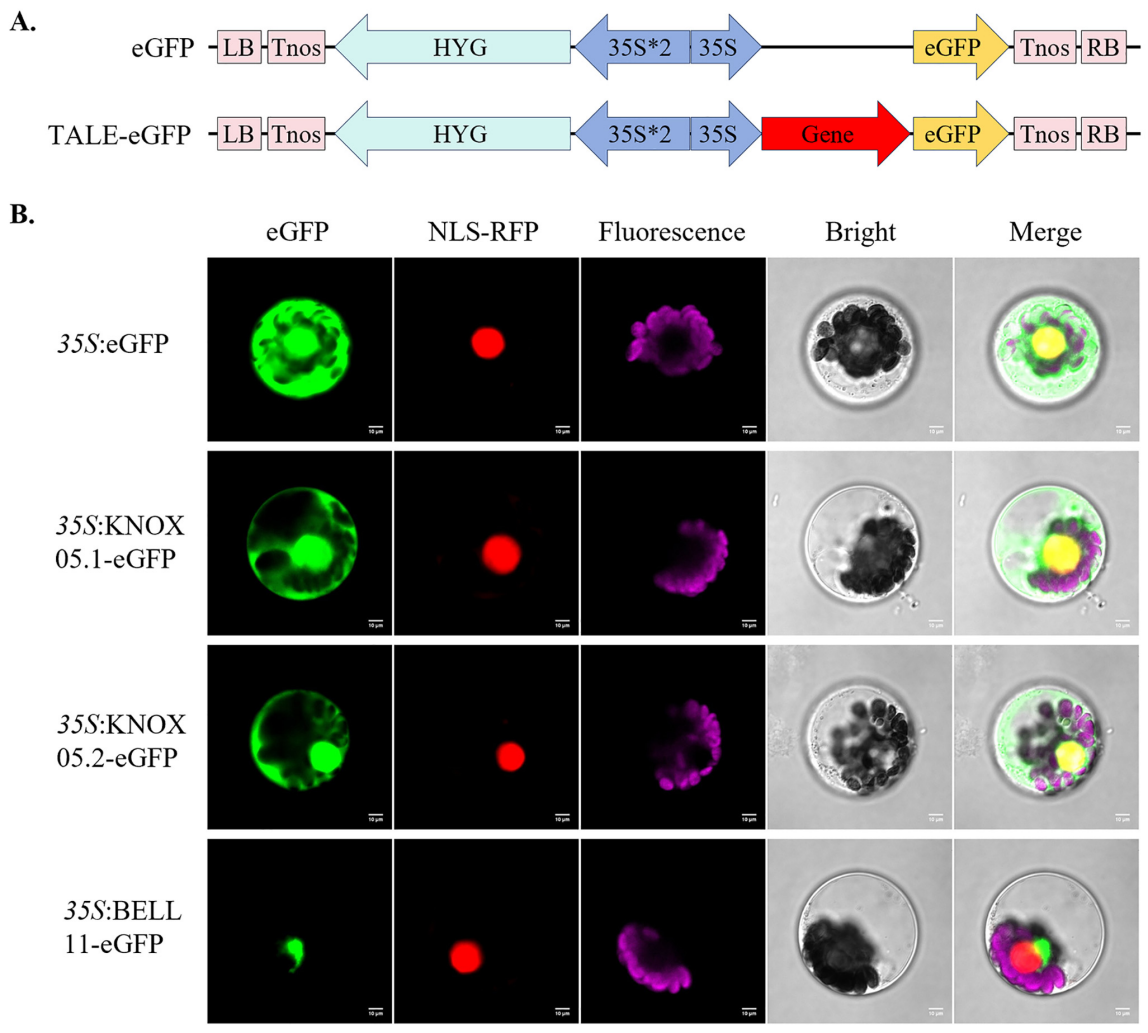
Subcellular localization of *ZmKNOX05.1*, *ZmKNOX05.2* and *ZmBELL11* in maize protoplasts. **(A)** Vector diagram of 35S:eGFP, 35S:ZmTALE-eGFP. **(B)** Fusion proteins were transiently expressed in maize protoplasts. 35S:eGFP vector was used as control, and *ZmKNOX05.1*, *ZmKNOX05.2* and *ZmBELL11*, fused with eGFP (green fluorescence). NLS-RFP (red fluorescence) is a marker for nucleus localization. Pink fluorescence belongs to chlorophyll in chloroplasts, merged fluorescence is in yellow. Bars = 10 µm. Images are representative of three independent experiments.

Subcellular localization of proteins is a critical step in understanding the cellular function of proteins. WoLF PSORT webserver predicted that all ZmTALE proteins were localized in the nucleus, in addition, BELL02, BELL07 and KNOX07 were also distributed in chloroplasts (**Supplementary Table 1**). To confirm these results, we selected the *ZmBELL11* in the BELL subfamily and the *ZmKNOX05* in the KNOX subfamily, which have relatively large changes in expression patterns after abiotic stress and ABA treatment, for subcellular localization analysis. *ZmKNOX05* and *ZmBELL11* were cloned from B73 maize, firstly. Unexpectedly, we discovered the existence of a novel transcript for *ZmKNOX05*, and named *ZmKNOX05.2*, which alternative splicing events at non canonical sites. The new alternative transcript differs from the published *ZmKNOX05.1* by a deletion of 99 nucleotides (33 amino acids), and its CDS and protein sequences were shown in **Supplementary Table 8**. Then, we constructed fusion proteins containing C-terminal enhanced green fluorescent protein (eGFP) (**Figure 13A**). The recombinant ZmKNOX05.1-eGFP, ZmKNOX05.2-eGFP, and ZmBELL11-eGFP proteins were transiently expressed in maize mesophyll protoplasts, respectively. The *35S*::eGFP used as the control, which was expressed in the nucleus and cytoplasm (**Figure 13B**). The results showed that the ZmKNOX05.1-eGFP, and ZmKNOX05.2-eGFP fusion were localized to the nucleus and cytoplasm, as confirmed by co-localization with the nucleus marker NLS. While ZmBELL11-eGFP partially co-localized with RFP-NLS, most of them were localized to the region around the RFP-NLS, indicating the perinuclear cytoplasmic region (**Figure 13B**). These results indicated that *ZmKNOX05* functions in the nucleus and cytoplasm, and *ZmBELL11* functions in perinuclear cytoplasm.

## Discussion

In recent years, TALE transcription factors have attracted much attention for their regulatory roles in plant growth, development, and abiotic stresses responses. Overexpression of *TaKNOX11* enhances plant resistance to salt and drought stress, and TaKNOX11-A can interact with six other BLH family members (Han et al., 2022). BLH1 over-expressing lines were hypersensitive to ABA and salinity, while *blh1* were less sensitive to ABA and salinity (Kim et al., 2013). And the BLH1-KNAT3 heterodimer increased the ABA responses by activating *ABI3* promoter (Kim et al., 2013). Although the TALE gene family has been recognized in multiple plant species and certain gene functions have been studied (Zhao et al., 2019; Wang et al., 2021; Han et al., 2022; Rathour et al., 2022; Yang et al., 2022; Liao et al., 2024), a comprehensive identification and functional analysis of TALE genes in maize, particularly under abiotic stress conditions such as heat, salt, and drought, as well as in response to ABA hormone treatment, has yet to be conducted. This research conducted a systematic analysis of the phylogeny, evolution, tissue expression profiles, stress responses, and subcellular localization of TALE genes in maize.

In the maize genome, 52 members of the TALE superfamily were identified and categorized into BELL and KNOX subfamilies according to their domain composition (see **Figures 1**, **2**), aligning with classifications observed in wheat, soybean, and barley (Wang et al., 2021; Han et al., 2022; Liao et al., 2024). This indicated that the TALE genes were highly conserved in the plant kingdom. Based on the results of phylogenetic relationship and chromosome localization analysis, we found that the ZmTALE genes on the same chromosome were also clustered together in phylogenetic tree. For example, all seven BELL genes located on chromosome 1 are members of the BELL2 subfamily, and all four KNOX genes belong to the KNOX I subfamily, except KNOX01 (**Figures 2**, **3**). These suggested that they may have evolved from gene duplication events. Consistently, within-species collinearity analysis revealed that ZmBELL01.1 and ZmBELL03.1, ZmBELL05.1 and ZmBELL07.2, and ZmKNOX02.2 and ZmKNOX03.3 were collinear gene pairs (**Figure 4**). Interestingly, most of the clustered genes also shared very similar expression patterns, as the case for *ZmBELL 2*/*3*/*6*/*7* under abiotic stress and ABA hormone, suggesting the clustered genes might be functionally redundant (**Figures 9**–**12**). This clustering indicates that gene duplication events might have played a role in the expansion and functional diversification of the ZmTALE genes.

Analysis of gene structure indicates that ZmKNOX family members possess a greater number of exons and introns, with introns being longer in comparison to ZmBELL. These structural variations may play a role in gene evolution and functional diversification (Roy and Gilbert, 2006). Different introns in ZmTALE genes may affect their evolution.

To further investigate the evolutionary process of ZmTALE members, we performed synteny analysis between maize, dicotyledons, and monocotyledons (**Figure 5**). The results showed that there were more ZmTALE homologs in the monocotyledons than in the dicots. This is similar to the conclusion of covariance analysis between species for the GmTALE genes in soybean (Wang et al., 2021). There were 18 ZmTALE genes with co-linear gene pairs in monocots but not in dicots, which distributed in both KNOX and BELL families, without subfamily specificity. So, there is not a subfamily of TALE genes that has undergone significant expansion or is unique in monocotyledons compared to dicotyledons. These 18 ZmTALE genes’ CDS sequences, protein motifs, and promoter *cis*-elements were also analyzed. Unfortunately, no similarity between them was found. Noticeably, ZmBELL08/11/13/14 and ZmKNOX03/04/06/09/11/12/13 had syntenic pairs in both dicotyledons and monocotyledons, suggesting that these gene pairs are conserved and may have existed before their ancestral divergence. The conservation of these gene pairs indicates that ZmTALE genes have preserved essential functions since the evolutionary split between monocots and dicots.

The *ZmTALEs* promoter contains a variety of plant growth and development, stress, and hormone responsive *cis*-elements (**Figure 7**), suggesting that they may be involved in plant responses to stress processes during plant growth and development. *KNAT 7* was expressed in seed coat, and interacted with MYB75 and other transcription factors in regulation of seed coat development in Arabidopsis (Bhargava et al., 2013). *ZmKNOX01* and *ZmKNOX14* were closely to *KNAT7* in evolutionary tree (**Figure 2**), which were highly expressed in the pericarp (**Figure 8**). We inferred that these two genes might be involved in pericarp development. This hypothesis needs to be verified by subsequent experiments. KNOX1 family gene *OSH15*, which was responsible for internode elongation by targeting a number of BR-related genes in rice (Niu et al., 2022). ZmKNOX1 subfamily genes were more highly expressed in the primordium, internode (**Figure 8**). We speculate that this family genes may regulate maize stem node growth and development through brassinosteroid signaling pathway. Although these hypotheses show promise, additional experimental work is essential to validate the involvement of ZmKNOX and ZmBELL genes in maize development via brassinosteroid signaling pathways. The qRT-PCR results showed that members of the *ZmTALEs* respond differentially to multiple abiotic stresses and stress-related hormone ABA. Overall, the expression of *ZmTALEs* were down-regulated under salt stress, drought stress and heat stress (**Figures 10**–**12**), and their expression was first up-regulated and then down-regulated after treatment with the stress hormone ABA (**Figure 9**). Its expression patterns under stress were similar to the previous researches in other species. In *Prunus mume*, four TALE genes were all downregulated under salt stress and drought stress (Yang et al., 2022). In wheat, *TaBLH4-D* showed a down-regulation under drought, salt, MeJA, and ABA stress (Han et al., 2022). And overexpression of *TaKNOX11* enhances drought and salt stress resistance in *Arabidopsis thaliana* by decreasing malondialdehyde content and increasing proline content (Han et al., 2022). There are 11 genes response to salt stress, and most of them down-regulated by salt stress in leaf in poplar (Zhao et al., 2019). *MtKNOX3-like* genes belonging to the KNOX2 family, enhance drought stress resistance in Arabidopsis by regulating the *MtPDH* gene to inhibiting proline degradation (Iannelli et al., 2023). *BLH1* over-expressing lines were hypersensitive to ABA and salinity, and BLH1 can interacted with KNAT3 and synergistically increased the ABA responses by binding to *ABI3* promoter (Kim et al., 2013). ABA can be involved in salt stress response through osmotic, ions, and reactive oxygen species (ROS) (Yu et al., 2020). Although exogenous ABA significantly induced the expression of *ZmTALEs* after 9h treatment, however, whether the functions of *ZmTALEs* were mediated through the ABA pathways is still unclear. The expression patterns of *ZmTALEs* in response to stress correspond with findings in other species, including *Prunus mume* and wheat, underscoring their likely conserved functions in abiotic stress resistance. Nevertheless, the regulatory mechanisms through ABA pathways require further exploration.

The KNOX family proteins include four typical domains: KNOX1, KNOX2, HD, and ELK domain. All ZmKNOX proteins contained these four domains, except ZmKNOX01 and ZmKNOX14 that lacked the ELK (**Figure 1C**). ELK domain spans about 21 amino acids, which is rich in glutamate (Glu, E), leucine (Leu, L), and lysine (Lys, K), and act as nuclear localization signal (NLS). KNOX proteins in Arabidopsis have both a putative NLS and an NES (nuclear export signal), suggested nucleo-cytoplasmic shuttling of these KNOXs (Kim et al., 2013). Despite containing the ELK domain, STM belonging to the KNOX1 family do not localize in the nucleus but in the cytoplasm (Cole et al., 2006). The fusion protein VAN-GFP, ATH1-GFP, GFP-BLH3, PNY-GFP fusion were found in both the cytosol and the nucleus (Bhatt et al., 2004; Cole et al., 2006). However, the VAN/STM, ATH1/STM, BLH3/STM and PNY/STM heterodimers were all become completely nuclear localized (Bhatt et al., 2004; Cole et al., 2006; Rutjens et al., 2009). Although, all ZmTALE proteins were localized in nucleus upon prediction of WOLF SPORT, we selected two genes *ZmBELL11* and *ZmKNOX05* for further investigated their subcellular localization. ZmKNOX05 was not only localized in the nucleus but also distributed in the cytoplasm. In contrast, ZmBELL11 was distributed in the perinuclear cytoplasm (**Figure 13**). KNOX proteins usually interact with BELL proteins to form KNOX-BELL heterodimers, which are necessary for their nuclear translocation and/or retention. We believe that KNOX-BELL interactions also exist in maize. Experimental evidence will be needed to show which ZmBELL members interact with ZmKNOX05, or which ZmKNOX members interact with ZmBELL11 to localize them in the nucleus. These interactions are essential for nuclear localization and are likely to affect the regulatory roles of ZmTALE proteins in maize. Subsequent investigations should focus on specific ZmKNOX-ZmBELL interactions to clarify their contributions to gene regulation.

Unexpected, a novel alternative transcript of ZmKNOX05, named ZmKNOX05.2 were found, which was deleted 99bp than published ZmKNOX05.1 transcript (**Supplementary Figure 4**). Secondary structure prediction using the SMART website (http://smart.embl-heidelberg.de/) revealed that ZmKNOX05.2 differs from ZmKNOX05.1 in low complexity at the N-terminal, with base deletions resulting in only one low-complexity domain at the N-terminal of ZmKNOX05.2 (**Supplementary Figure 5**). The missing 33 amino acids is rich in alanine. SWISS-MODEL website (https://swissmodel.expasy.org/) predicted the 3D structures of the proteins and showed that ZmKNOX05.2 has one less section of irregular coiling than ZmKNOX05.1 at the N-terminus (**Supplementary Figure 5**). Alternative splicing enriches the diversity of genes and proteins functions. KNOX2 transcription factors *HOS58*, *HOS59*, and *HOS66*, produce two alternative transcripts in rice embryos (Ito et al., 2002), and HOS58 orthologue of KNOX7 in maize also has two alternative transcripts in embryos due to differential transcription initiation (Morere-Le-Paven et al., 2007). KNOX7S and/or the ratio of KNOX7S/KNOX7L regulate seed germination rates through the ABA/GA signaling pathways (Morere-Le-Paven et al., 2007). To date, the presence of noncanonical alternative splicing events in KNOX1 transcription factors has not been reported. Although ZmKNOX05.2 and ZmKNOX05.1 were both localized in the nucleus and cytoplasm, the absence of this irregular coiling may result in different functions of ZmKNOX05. What is the splicing mechanism of ZmKNOX05, and what is the function of ZmKNOX05.1 and ZmKNOX05.2, need to be answered in the future.

## Conclusion

We identified 52 TALE superfamily members in maize, which were unevenly distributed on 9 chromosomes. They can be divided into two subfamilies, the BELL and the KNOX. Besides, the KNOX subfamily was separated into KNOX1 and KNOX2 subclasses. And the BELL subfamily was separated into BELL1 and BELL2 subclasses. Gene structures, conserved domain and motif compositions of the ZmTALE members in the same subfamily was similar, whereas, varied greatly in different subfamily. ZmKNOX family members possess a greater number of exons and introns but smaller molecular weights and greater hydrophilicity than ZmBELL. And the KNOX proteins included KNOX1, KNOX2, HD, and ELK, four typical domains, while BELL proteins contained POX and HD domains. The syntenic and evolutionary analyses of the TALE proteins among maize and multiple species provided more detailed evidence for ZmTALE genes evolution. *Cis*-elements analyses in gene promoter regions, as well as quantitative RT-PCR detection, showed that the *ZmTALEs* play important roles during maize growth and development, and abiotic stress responses. MYB, STRE, and ABRE *cis*-elements are abundant on the promoters of *ZmTALEs*. Most *ZmTALEs* were down-regulated under salt, drought, and heat stress, while their expression was first up-regulated and then down-regulated after ABA treatment. Subcellular localization analysis revealed that ZmKNOX05.1 and its alternative spliced form ZmKNOX05.2 were localized in both the nucleus and cytoplasm, while ZmBELL11 was localized in perinuclear cytoplasm. Our work revealed several important aspects of ZmTALE family members, which lay a foundation for the functional study of *ZmTALEs* in the future. The discovery of ZmKNOX05.2 sheds light on alternative splicing events within the KNOX1 family, indicating possible functional diversification. Future studies should delve into its splicing mechanisms and the distinct functions it may serve in comparison to ZmKNOX05.1.

## Data Availability

The datasets presented in this study can be found in online repositories. The names of the repository/repositories and accession number(s) can be found in the article/**Supplementary Material**.

## Supplementary material

Supplementary Figure 1 Comparison of maize TALE protein sequences by MegAlin. All ZmTALEs contained PYP amino acid sequence, which was highlighted in yellow in the figure.

Supplementary Figure 2 Correspondence and number statistics of covariant gene pairs between maize and mono-dicotyledonous plants. **(A)** Heat map of the correspondence and number of covariant gene pairs between maize and mono-dicotyledonous plants. Colors from blue to red indicated the number from 0 to 6. **(B)** Summary plots of the types and number of covariant gene pairs in **(A)**. **(C)** Correspondence of covariant gene pairs between maize and monocotyledons (blue round ball), dicotyledons (black squares), and within the maize genome (red triangles).

Supplementary Figure 3 Eight motifs of the TALE proteins predicted by the MEME website.

Supplementary Figure 4 The protein sequences of ZmKNOX05.1 and ZmKNOX05.2 compared by DNAMAN.

Supplementary Figure 5 Secondary and tertiary structure of ZmKNOX05.1 and ZmKNOX05.2 predicted by SMART and SWISS-MODEL. **(A)** Secondary and tertiary structure of ZmKNOX05.1. **(B)** Secondary and tertiary structure of ZmKNOX05.2. The pink boxes in the secondary structure represent low complexity. A in the tertiary structure represents amino acid and the number represents the amino acid position.

Supplementary Table 8 The CDS and protein sequence of ZmKNOX05.1 and novel transcript ZmKNOX05.2, respectively.
