# Supplementary figures and images for: Genome-wide identification and expression profile analysis of TALE superfamily genes under hormone and abiotic stress in maize (Zea may L.)
Source: Front Plant Sci. 2025 Feb 21;16:1489177. doi: 10.3389/fpls.2025.1489177 (PMC11885238; doi:10.3389/fpls.2025.1489177)

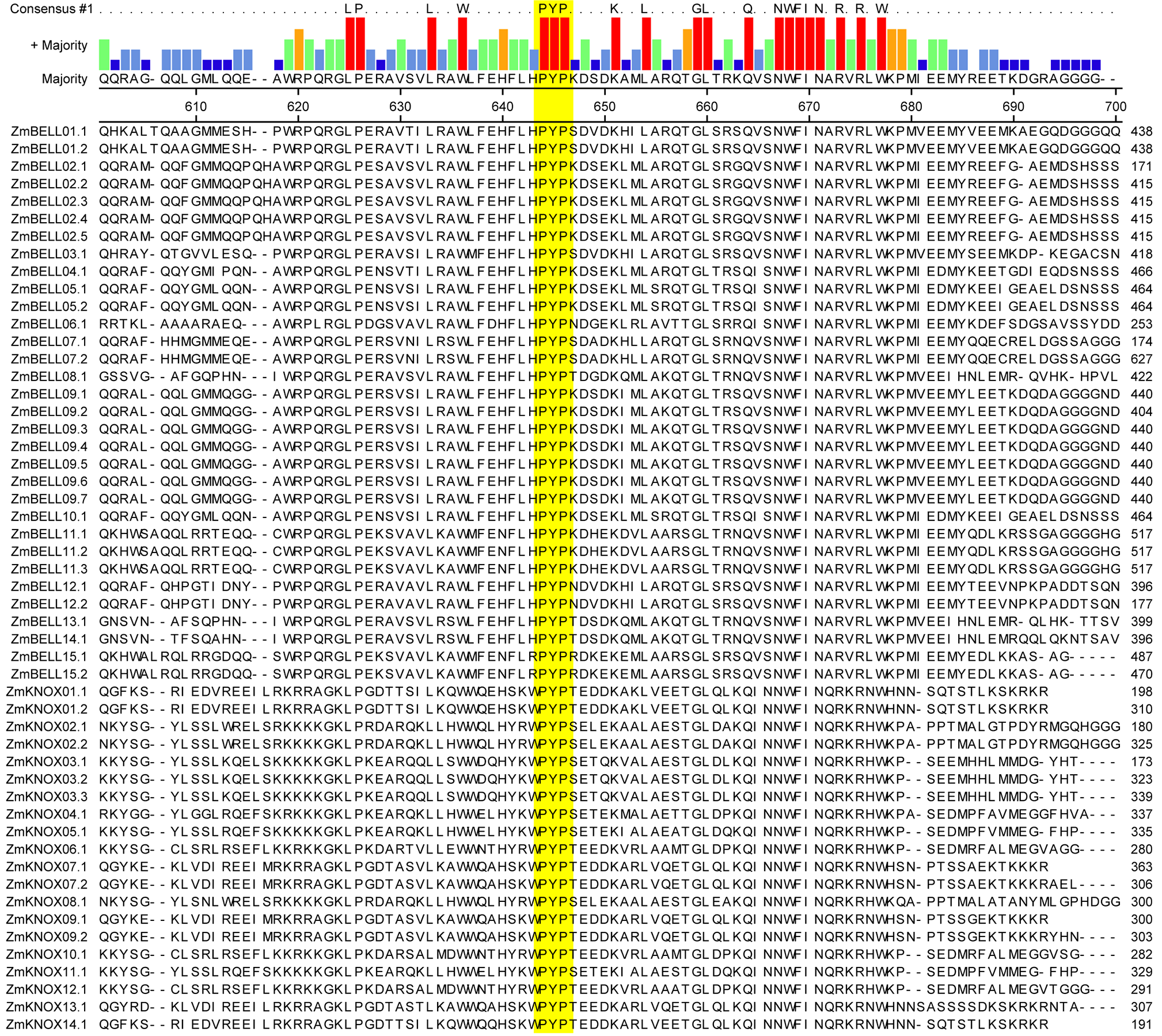

Supplement: Supplementary Figure 1 — Comparison of maize TALE protein sequences by MegAlin. All ZmTALEs contained PYP amino acid sequence, which was highlighted in yellow in the figure. [file Image1.png]

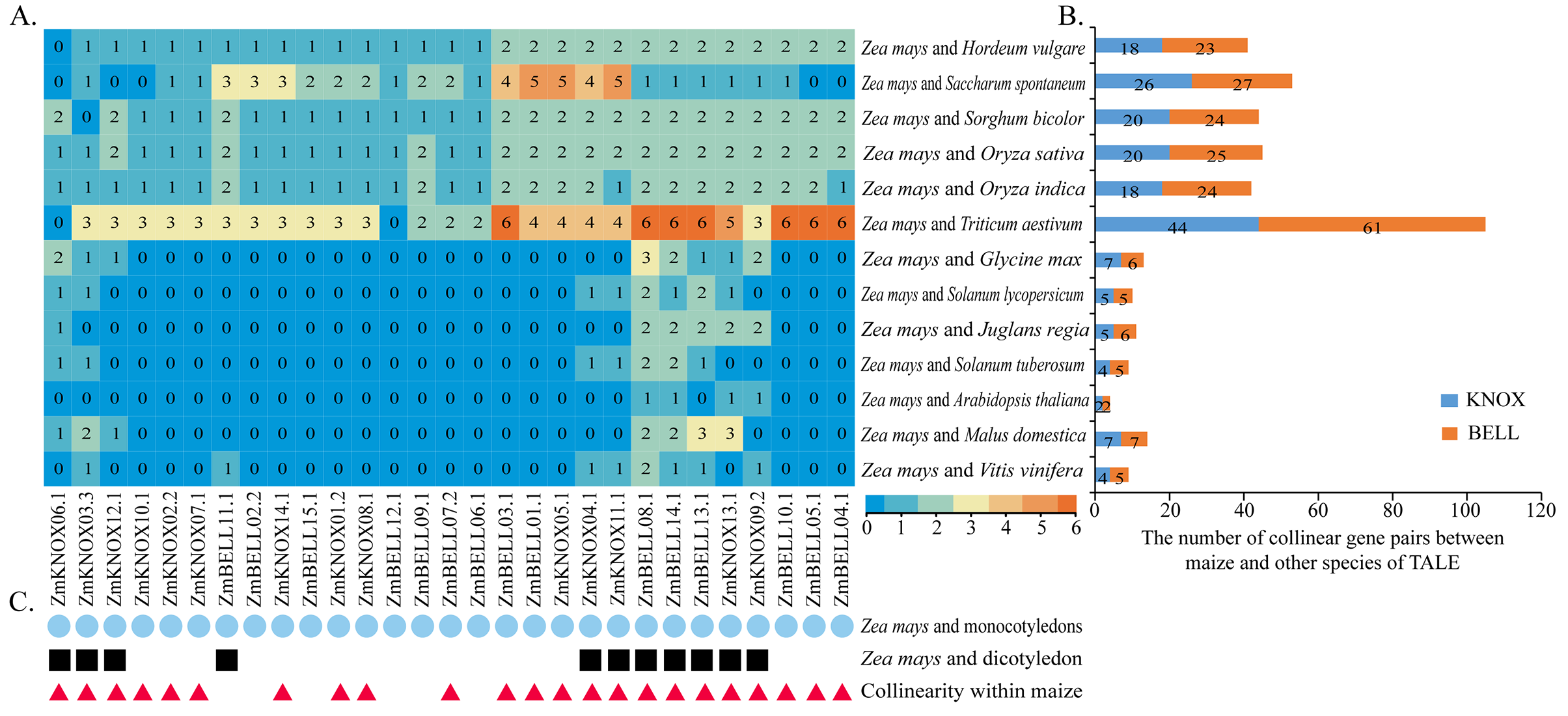

Supplement: Supplementary Figure 2 — Correspondence and number statistics of covariant gene pairs between maize and mono-dicotyledonous plants. (A) Heat map of the correspondence and number of covariant gene pairs between maize and mono-dicotyledonous plants. Colors from blue to red indicated the number from 0 to 6. (B) Summary plots of the types and number of covariant gene pairs in (A). (C) Correspondence of covariant gene pairs between maize and monocotyledons (blue round ball), dicotyledons (black squares), and within the maize genome (red triangles). [file Image2.png]

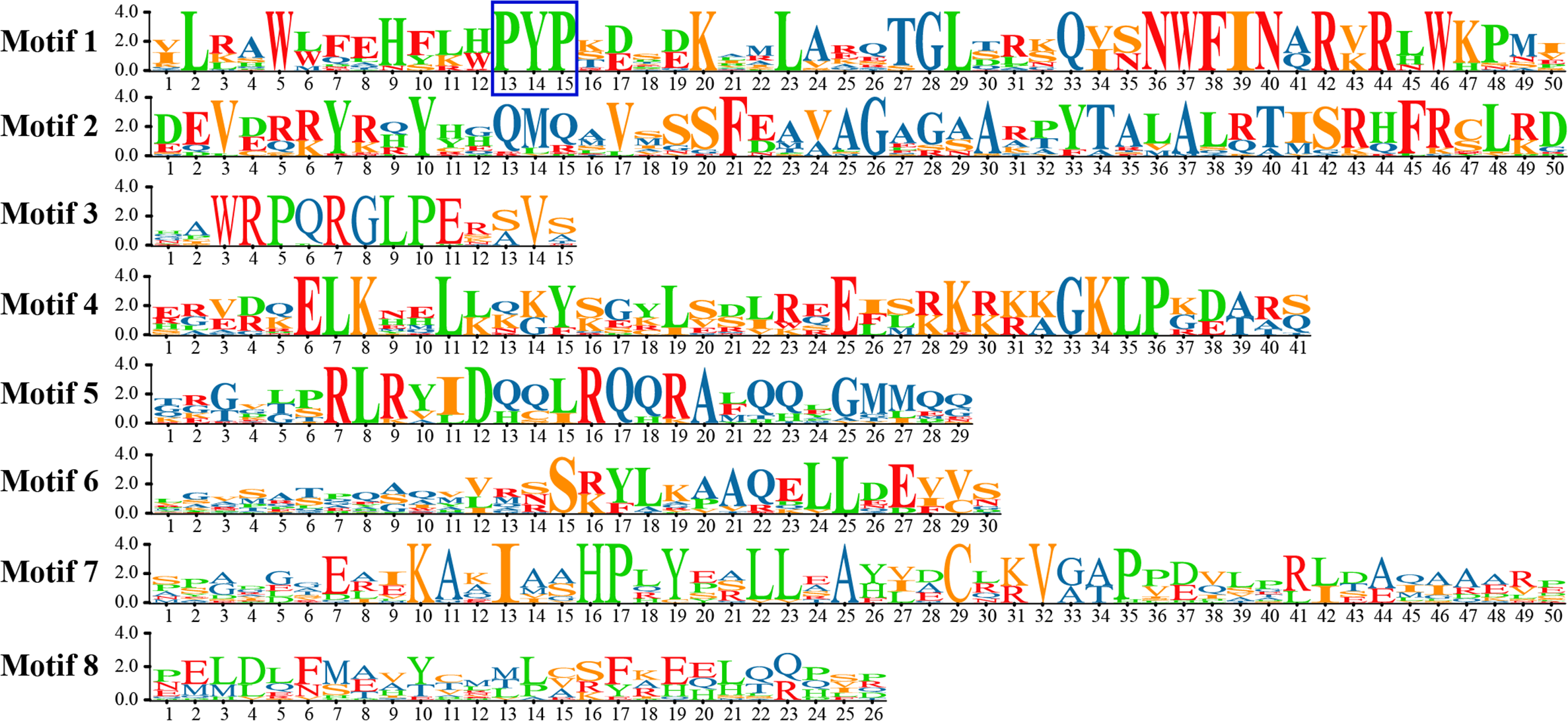

Supplement: Supplementary Figure 3 — Eight motifs of the TALE proteins predicted by the MEME website. [file Image3.png]

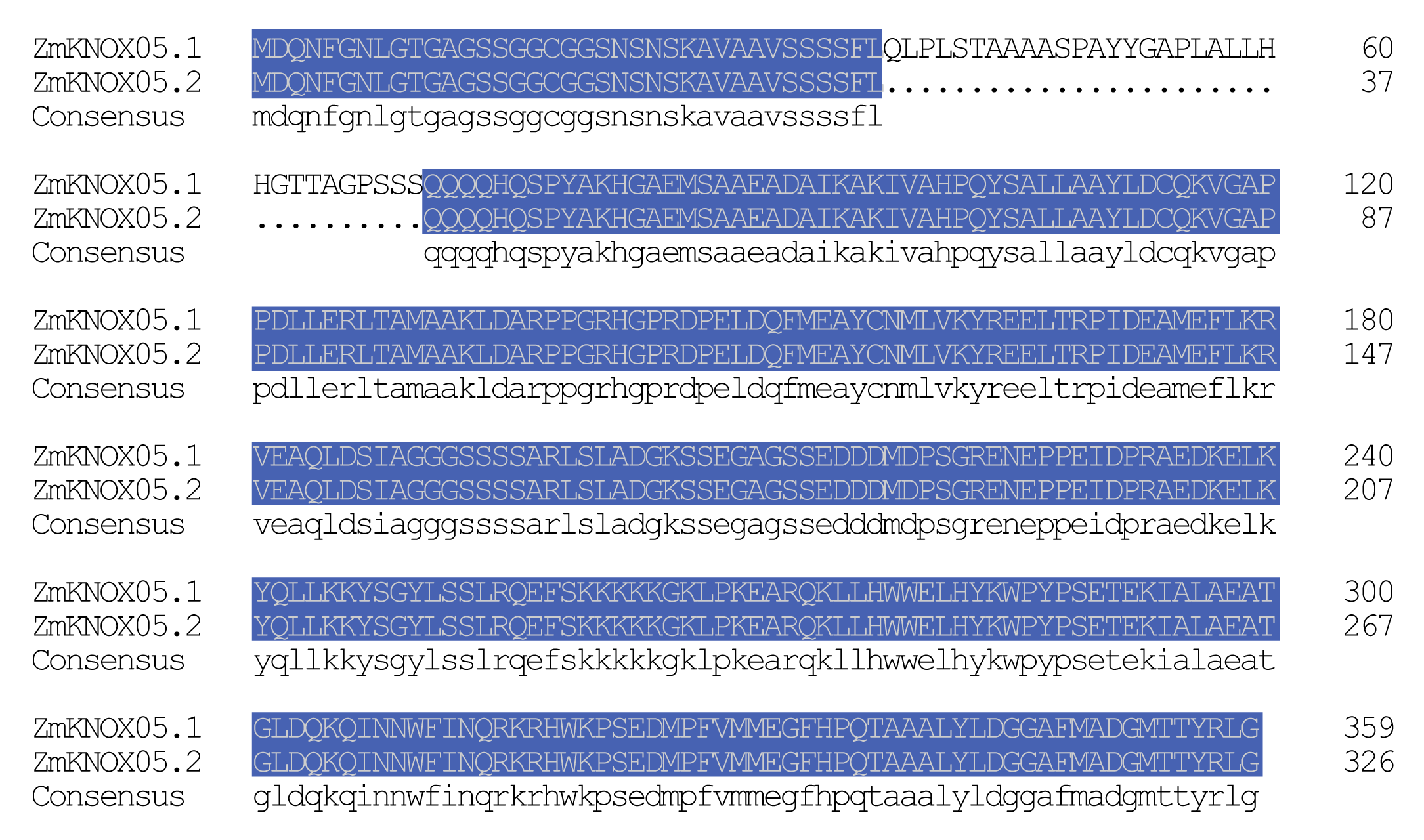

Supplement: Supplementary Figure 4 — The protein sequences of ZmKNOX05.1 and ZmKNOX05.2 compared by DNAMAN. [file Image4.png]

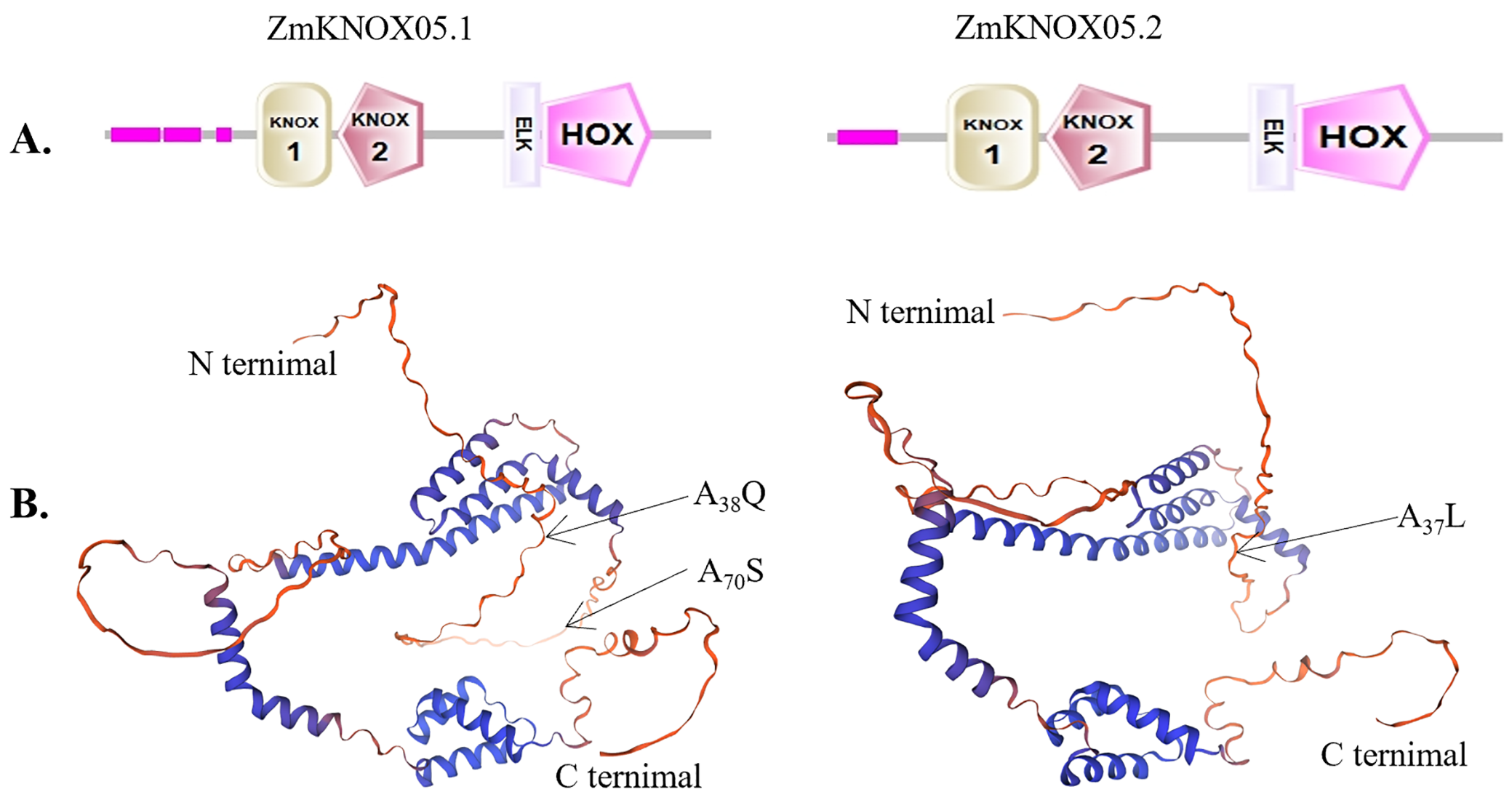

Supplement: Supplementary Figure 5 — Secondary and tertiary structure of ZmKNOX05.1 and ZmKNOX05.2 predicted by SMART and SWISS-MODEL. (A) Secondary and tertiary structure of ZmKNOX05.1. (B) Secondary and tertiary structure of ZmKNOX05.2. The pink boxes in the secondary structure represent low complexity. A in the tertiary structure represents amino acid and the number represents the amino acid position. [file Image5.png]
